# Contribution of common and rare variants to Asian neovascular age-related macular degeneration subtypes

**DOI:** 10.1038/s41467-023-41256-z

**Published:** 2023-09-11

**Authors:** Qiao Fan, Hengtong Li, Xiaomeng Wang, Yih-Chung Tham, Kelvin Yi Chong Teo, Masayuki Yasuda, Weng Khong Lim, Yuet Ping Kwan, Jing Xian Teo, Ching-Jou Chen, Li Jia Chen, Jeeyun Ahn, Sonia Davila, Masahiro Miyake, Patrick Tan, Kyu Hyung Park, Chi Pui Pang, Chiea Chuan Khor, Tien Yin Wong, Yasuo Yanagi, Chui Ming Gemmy Cheung, Ching-Yu Cheng

**Affiliations:** 1 Center for Quantitative Medicine, Duke-NUS Medical School, National University of Singapore, Singapore, Singapore; 2 Ophthalmology & Visual Sciences Academic Clinical Program (Eye ACP), Duke-NUS Medical School, Singapore, Singapore; 3 Singapore Eye Research Institute, Singapore National Eye Centre, Singapore, Singapore; 4 Department of Ophthalmology, Yong Loo Lin School of Medicine, National University of Singapore, Singapore, Singapore; 5 Centre for Innovation and Precision Eye Health, Yong Loo Lin School of Medicine, National University of Singapore, Singapore, Singapore; 6 Center for Vision Research, Duke-NUS Medical School, National University of Singapore, Singapore, Singapore; 7 Department of Ophthalmology, Tohoku University Graduate School of Medicine, Miyagi, Japan; 8 SingHealth Duke-NUS Institute of Precision Medicine, Singapore, Singapore; 9 SingHealth Duke-NUS Genomic Medicine Centre, Singapore, Singapore; 10 Cancer and Stem Cell Biology Program, Duke-NUS Medical School, Singapore, Singapore; 11 Laboratory of Genome Variation Analytics, Genome Institute of Singapore, Agency for Science, Technology and Research, Singapore, Singapore; 12 Department of Ophthalmology and Visual Sciences, The Chinese University of Hong Kong, Hong Kong, China; 13 Department of Ophthalmology, Seoul National University College of Medicine, Seoul Metropolitan Government Seoul National University Boramae Medical Center, Seoul, Korea; 14 Department of Ophthalmology and Visual Sciences, Kyoto University Graduate School of Medicine, Kyoto, Japan; 15 Human Genetics, Genome Institute of Singapore, Singapore, Singapore; 16 Department of Ophthalmology, Seoul National University Hospital, Seoul, Korea; 17 Tsinghua Medicine, Tsinghua University, Beijing, China; 18 Department of Ophthalmology and Microtechnology, Yokohama City University, Yokohama, Japan

**Keywords:** Genetic association study, Macular degeneration

## Abstract

Neovascular age-related macular degeneration (nAMD), along with its clinical subtype known as polypoidal choroidal vasculopathy (PCV), are among the leading causes of vision loss in elderly Asians. In a genome-wide association study (GWAS) comprising 3,128 nAMD (1,555 PCV and 1,573 typical nAMD), and 5,493 controls of East Asian ancestry, we identify twelve loci, of which four are novel ($P \, < \, 1.19\times {10}^{-8}$). Substantial genetic sharing between PCV and typical nAMD is noted (*r*_*g*_ = 0.666), whereas collagen extracellular matrix and fibrosis-related pathways are more pronounced for PCV. Whole-exome sequencing in 259 PCV patients revealed functional rare variants burden in collagen type I alpha 1 chain gene (*COL1A1*; $P=1.05\times {10}^{-6}$) and potential enrichment of functional rare mutations at AMD-associated loci. At the GATA binding protein 5 *(GATA5)* locus, the most significant GWAS novel loci, the expressions of genes including laminin subunit alpha 5 (*Lama5)*, mitochondrial ribosome associated GTPase 2 (*Mtg2)*, and collagen type IX alpha 3 chain (*Col9A3)*, are significantly induced during retinal angiogenesis and subretinal fibrosis in murine models. Furthermore, retinoic acid increased the expression of *LAMA5* and *MTG2* in vitro. Taken together, our data provide insights into the genetic basis of AMD pathogenesis in the Asian population.

## Abstract (web summary)

A deep understanding of the genetic signatures of neovascular age-related macular degeneration subtypes in Asian patients remains a significant gap. Here the authors pinpoint contributing common and rare variants using GWAS and exome sequencing approaches.

## Introduction

Age-related macular degeneration (AMD) is one of the leading causes of irreversible vision loss among people aged 50 years and above. By 2040, more than 170 million people are estimated to be affected by this debilitating eye disease, with over half of the incidence in Asia alone, largely due to its rapidly aging population^1^. Neovascular AMD (nAMD) is a late-stage AMD, characterized by exudation, subretinal hemorrhage, and scarring, which often lead to central vision loss^2,3^. In Asian populations, polypoidal choroidal vasculopathy (PCV) is a common subtype of overall nAMD. PCV accounted for up to $55\%$ of all nAMD in Asian populations^4–6^, compared to less than $10\%$ of counterparts in white populations^7,8^. Although they share many common features, PCV is a distinct phenotype of typical nAMD. Clear differences in morphological and pathogenic features exist: PCV develops in eyes with “pachychoroid” and is not necessarily associated with drusen, subretinal pigment epithelial deposits^3,9^. On the other hand, drusen are the most salient features of typical nAMD. Clinically, PCV patients are generally younger and may present with recurrent episodes of sub-macular hemorrhage, possibly with less scarring and variations in their response to anti-VEGF therapy compared to typical nAMD patients^10,11^. Hence, a deep understanding of the biology and genetic signatures of PCV and typical nAMD is important for the identification of new drug targets that are more effective in treating Asian patient populations.

Some earlier studies have identified common and rare variants that contribute to the genetic architecture of AMD. However, most large-scale genome-wide scans were conducted in European populations, and data for the Asian AMD patient population, in particular for PCV, are scarce. Genome-wide association studies (GWAS) have identified ~51 loci associated with early or advanced AMD^12–15^. The association of several of these loci with PCV has been confirmed using candidate genes or meta-analysis approaches, such as *CFH*, *ARMS2*/*HTRA1*, *C2/CFB/SKIV2L*, *CETP*, and *VEGFA*, etc.^16–19^. Although these loci are shared between typical nAMD and PCV, the lead SNP may exhibit varying effect sizes. For instance, *ARMS2*/*HTRA1* variants (e.g., rs10490924, rs2672598) have a larger effect in typical nAMD than in PCV^19,20^. Furthermore, several rare functional variants, mainly involving complement factors-related genes such as *CFH* (e.g., p.Arg1210Cys, p.Arg303Gln), *C9* (e.g., p.Pro167Ser), *C3* (e.g., p.Lys155Gln), *CFI* (e.g., p.Gly119Arg)^21–23^, *CETP* (e.g., p.Arg303Gln)^13^, *SPEF2* (e.g., p.Arg201Gly)^24^ and *UBE3D* (rs7739323,1039G > A)^25^, have been reported to be associated with nAMD. Additionally, an earlier whole-genome exome (WES) study showed that rare variants in *FGD6* (p.Lys329Arg) are associated with PCV in the Chinese population^26^. Thus, a comprehensive study is needed to further identify susceptibility genes for PCV and typical nAMD in the Asian population, and to differentiate potential genetic heterogeneity between the two conditions.

In this work, we seek to characterize the genetic architecture underlying PCV and typical nAMD through GWAS and WES approaches in East Asian samples from the Genetics of AMD in Asian (GAMA) Consortium. Our study, to our knowledge, is the most comprehensive GWAS meta-analysis comprising 1555 PCV, 1573 typical nAMD and 5493 healthy controls from Singapore, Hong Kong, Korea and Japan, and 259 PCV sequenced samples. We identify novel loci shared between PCV and typical nAMD, potential causal genes within these loci, as well as rare functional variant enrichment that possibly confers genetic susceptibility to Asian PCV and typical nAMD pathogenesis.

## Results

### Novel GWAS loci identified for Asian nAMD, PCV and typical AMD

The detailed information on patients of East Asian ancestry in participating studies from the GAMA Consortium is described in “Methods” and Supplementary Notes. Supplementary Fig. 1 depicts a flow chart of the participants recruited in each study and included for analysis, while Supplementary Data 1 represents information on AMD phenotyping and genotyping arrays. At the study level, stringent quality control (QC) was performed before imputation on the genotyped markers (see “Methods”). For each study, the genotype data were imputed using the 1000 Genomes Project multi-ethnic reference panels v3 on overlapped SNPs across genotyping arrays in cases and controls. Principal component analysis (PCA) showed that cases and controls were genetically well-matched (Supplementary Fig. 2).

For the discovery phase, meta-analyses were conducted in 8,560,176 variants in 1687 nAMD patients and 3437 controls of Chinese ancestry from Singapore and Hong Kong studies, using fixed-effect models implemented in the METAL software (http://csg.sph.umich.edu/abecasis/Metal/download/). The quantile-quantile (QQ) plots showed little evidence of inflation in test statistics due to the population stratification (Supplementary Fig. 3; Genomic Control ${\lambda }_{{GC}}$: 0.987). About 3180 variants with *P* values less than $5\times {10}^{-5}$ at the discovery stage were carried over to the replication phase, for replicating in samples comprising 1441 nAMD patients and 2056 controls recruited from Korean and Japanese studies. Among these variants, 11 loci were identified with lead SNPs exceeding genome-wide significance (Supplementary Data 2A). We further performed a whole-genome meta-analysis of four cohorts for PCV and typical nAMD separately and identified an additional locus *NEK6/LHX2* for typical nAMD (Supplementary Data 2B). Our final sample for meta-analysis included 1555 PCV and 1573 typical nAMD patients, and 5493 healthy controls.

#### *GATA5/LAMA5* is the top novel locus for Asian nAMD

**Fig. 1:**
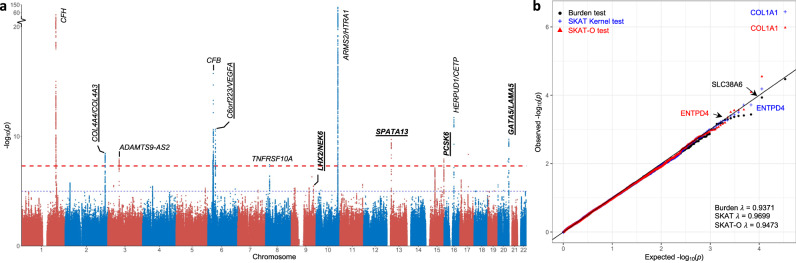
Manhattan plot of Manhattan plot of GWAS for neovascular AMD (nAMD) and QQ plot of whole-exome sequencing gene-level analysis for PCV. **a** We conducted genome-wide single-variants association analyses for nAMD in 3128 patients (1555 PCV and 1573 typical nAMD) and 5493 controls. The Manhattan plot exhibits ten logarithms of *P* values for the association, annotating the nearest genes for each genetic locus that reaches genome-wide significance ($P\, < \,5\times {10}^{-8}$). The gene names of novel nAMD loci are highlighted in bold. Two loci (*COL4A4/COL4A3* and *C6orf223/VEGFA*) that showed significant association with PCV in our study are underlined. The red dashed horizontal line represents genome-wide significance ($P\, < \,5\times {10}^{-8}$), and the blue line indicates the suggestive significance of $P\, < \,1\times {10}^{-5}$. The *y*-axis breaks at around $P=1\times {10}^{-20}$. **b** We conducted three gene-based analyses: Burden test, Sequence Kernel Association Test (SKAT), and Combined test of burden test and SKAT (SKAT-O) for functional variants at MAF $< 2\%$ in 1019 Chinese subjects (259 PCV and 760 controls). Data points shown with distinct hues and shapes represent genes tested for three different tests. The genomic inflation factor ($\lambda$) of each analysis is shown in the figure.

Altogether, we identified 12 loci with genome-wide significance; of these four were novel, including *GATA5/LAMA5* at Chromosome 20q13.33*, SPATA13* at Chromosome 13q12.12*, PCSK6* at Chromosome 15q26.3, and *NEK6/LHX2* at Chromosome 9q33.3 (Manhattan plot, Fig. 1a; Regional plots, Supplementary Fig. 4). Except for the *NEK6/LHX2* locus, signals at the rest of the loci were shared between PCV and typical nAMD. The strongest signal was at *GATA5/LAMA5* for nAMD (lead SNP rs6121609; $P=1.80\times {10}^{-10}$), whereas consistent effects were observed for AMD subtypes (rs6121609 C allele; PCV, ${{{{{{\rm{OR}}}}}}}=1.44$, $P=1.97\times {10}^{-7}$; typical nAMD, ${{{{{{\rm{OR}}}}}}}=1.45$, $P=1.34\times {10}^{-7}$; Table 1). Similarly, at the *PCSK6* and *SPATA13* loci, the magnitude of effects in all samples (*PCSK6* rs7402624 C allele; ${{{{{{\rm{OR}}}}}}}=1.22$, $P=1.19\times {10}^{-8}$; *SPATA13* rs4769312 A allele; ${{{{{{\rm{OR}}}}}}}=1.29$, $P=1.90\times {10}^{-10}$) were concordant across subtypes. The signal at the *NEK6/LHX2* locus, was mainly driven by typical nAMD (rs72759285 A allele; PCV, ${{{{{{\rm{OR}}}}}}}=1.10$, $P=6.84\times {10}^{-2}$; typical nAMD, ${{{{{{\rm{OR}}}}}}}=1.39$, $P=1.19\times {10}^{-8}$). Conditional analysis revealed two independent low-frequent variants at the known *CFH* locus (MAF$\le 6.3\%$; Supplementary Data 3). No additional novel loci were identified from the gene-based test using MAGMA (Supplementary Data 4).

**Table 1:** Association results for novel genetic loci for Asian nAMD and subtypes

Lead variant Chr:Position Nearest gene EA/OA EAF (PCV/typical nAMD/control)	rs72759285^a^ 9:126985763 *NEK6/LHX2* A/G 0.80/0.84/0.80			rs4769312 13:24623220 *SPATA13* A/G 0.76/0.77/0.72			rs7402624 15:102033538 *PCSK6* C/T 0.55/0.55/0.50			rs6121609 20:61038322 *GATA5/LAMA5* C/T 0.16/0.16/0.13		
	OR (95% CI)	*P*	Het *I*^2^	OR (95% CI)	*P*	Het *I*^2^	OR (95% CI)	*P*	Het *I*^2^	OR (95% CI)	*P*	Het *I*^2^
Discovery (*n* = case/control: 1687/3437)	1.20 (1.06, 1.34)	$2.59\times {10}^{-3}$		1.26 (1.14, 1.41)	$1.29\times {10}^{-5}$		1.21 (1.10, 1.32)	$3.69\times {10}^{-5}$		1.47 (1.27, 1.70)	$1.94\times {10}^{-7}$	
Replication (1441/2056)	1.26 (1.11, 1.43)	$3.27\times {10}^{-4}$		1.32 (1.17, 1.48)	$2.99\times {10}^{-6}$		1.24 (1.11, 1.37)	$7.90\times {10}^{-5}$		1.38 (1.16, 1.63)	$1.94\times {10}^{-4}$	
Meta-analysis for nAMD (3128/5493)	1.23 (1.13, 1.34)	$3.36\times {10}^{-6}$	0	1.29 (1.19, 1.39)	$1.90\times {10}^{-10}$	0	1.22 (1.14, 1.31)	$1.19\times {10}^{-8}$	0	1.43 (1.28, 1.60)	$1.80\times {10}^{-10}$	20.8
Meta-analysis for PCV (1555/5493)	1.10 (0.99, 1.23)	$6.84\times {10}^{-2}$	0	1.24 (1.12, 1.37)	$2.62\times {10}^{-5}$	0	1.24 (1.14, 1.35)	$1.44\times {10}^{-6}$	0	1.44 (1.26, 1.66)	$1.97\times {10}^{-7}$	0
Meta-analysis for typical nAMD (1573/5493)	1.39 (1.24, 1.56)	$1.19\times {10}^{-8}$	0	1.34 (1.21, 1.48)	$1.74\times {10}^{-8}$	0	1.20 (1.10, 1.30)	$5.48\times {10}^{-5}$	0	1.45 (1.26, 1.66)	$1.34\times {10}^{-7}$	20.4
Independent variants for PCV at known loci												
Lead variant	Chr: Position	Nearest gene	PCV (1555/5493)			typical nAMD (1573/5493)			EA/OA		EAF	
			OR (95% CI)	*P*	Het *I*^2^	OR (95% CI)	*P*	Het *I*^2^				
rs56033528	2:227865660	*COL4A4/COL4A3*	1.42 (1.27, 1.59)	$7.01\times {10}^{-10}$	0	1.19 (1.06, 1.34)	$2.63\times {10}^{-3}$	0	A/G		0.20/0.17/0.15	
rs73733647	6:43961015	*C6orf223/VEGFA*	1.49 (1.31, 1.69)	$8.33\times {10}^{-10}$	0	1.32 (1.16, 1.49)	$1.33\times {10}^{-5}$	67.3	G/A		0.83/0.82/0.79	

*Chr* chromosome, *EA* effect allele, *OA* other allele, *EAF* effect allele frequency, *OR (95% CI)* odds ratio (95% confidence Interval), *Het I*^*2*^ fixed-effect meta-analysis heterogeneity I-square statistics, *nAMD* neovascular AMD, *PCV* polypoidal choroidal vasculopathy.

Physical positions and nearest genes are based on NCBI build 37 of the human genome. Discovery stage: Chinese samples from Singapore and Hong Kong studies; replication stage: samples from Korea and Japan studies. We conducted a genome-wide single-variant association analysis using the firth bias-corrected likelihood-ratio model, incorporating the top four principal components as covariates. ${\chi }^{2}$ statistics with one degree-of-freedom was used for generating *P* values. We performed a meta-analysis under an inverse-weighted fixed-effects model across all cohorts for each phenotype.

^a^SNP rs72759285 at locus *NEK6/LHX2* is genome-wide significant ($1.19\times {10}^{-8}$) in GWAS for typical nAMD.

For those previously identified GWAS loci for advanced AMD in European populations^12,14^, Supplementary Data 5 listed the association between index SNPs and nAMD in Asian samples. Among 37 genetic loci, lead variants at 4 loci reached genome-wide significance ($10.8\%$; $P\, < \,5\times {10}^{-8}$); 17 had nominal significance ($45.9\%$; $5\times {10}^{-8}\, < \,P\, < \,0.05$), and 16 did not show an association ($43.2\%$; $P > 0.05$). For those lead variants not showing genome-wide significance, the genetic effects were moderate, in general with an odds ratio (OR) less than 1.20. Across all these variants, the correlation between beta effects from our datasets and those reported from European samples was 0.831 ($P\, < \,1.90\times {10}^{-10}$).

#### Independent variant in *COL4A4* and *C6orf223* genes associated with PCV

The following five genome-wide loci in decreasing order of significance for PCV are also known loci for nAMD: *ARMS2/HTRA1, CFH, COL4A4/COL4A3, C6orf223/VEGFA, C2/CFB*, and *CETP*. For the *COL4A4/COL4A3* locus at chromosome 2, we identified a lead SNP rs56033528 in gene *COL4A4* for PCV ($P=7.01\times {10}^{-10}$), with an attenuated effect for typical nAMD ($P=2.63\times {10}^{-3}$). SNP rs56033528 is independent (${r}^{2}=0.002$) of the previously identified lead SNP rs11884770^12^ at the *COL4A3* locus for advanced AMD in European populations (Table 1). Previously, we identified the *C6orf223/VEGFA* locus for nAMD from our GAMA GWAS data^13^. In this study, the lead SNP rs73733647 at *C6orf223/VEGFA* reached genome-wide significance for PCV ($P=8.33\times {10}^{-10}$). SNP rs73733647 is independent of the lead SNP rs943080 at the *VEGFA* locus identified in the European population (${r}^{2}\, < \,0.001$).

#### Transethnic-replication in European populations

Next, we evaluated the transethnic replication of the above 6 loci in individuals of European ancestry from the International AMD Genomics Consortium (IAMDGC) dataset (16,144 cases and 17,832 controls). Lead SNPs at *GATA5/LAMA5, PCSK6*, and *COL4A4* showed potential association with AMD in this dataset ($P\, < \,0.05$ ; Supplementary Data 6). Overall, the directions of effect sizes were largely similar.

### WES pinpoints potential genes of rare variants’ enrichment for PCV

To reveal any potential functional variants for PCV that alter peptide sequences (missense), or lead to stop-gain, stop-loss, frameshifting, or splice-site disruption, we performed exome sequencing in 259 PCV patients and 760 controls of the Chinese ancestry population in Singapore. Following the standard GATK pipeline (https://github.com/broadinstitute/gatk/releases/tag/4.0.3.0), we detected 637,799 single-nucleotide variants (SNVs), of which 166,045 were functional. In both patient samples and controls, there was substantial concordance between genotypes called by exome sequencing and the directly genotyped data from GWAS, for variants with MAF at the following ranges: $5-50\%$ (concordance $> 99\%$), $1-5\%$ (concordance $> 98\%$), $< 1\%$ (concordance $> 95\%$; Supplementary Fig. 5). We conducted gene-based tests of the kernel (SKAT) and burden, and omnibus SKAT-O combining both, for PCV in 154,195 functional variants with MAF $\le 2\%$. We used the SKAT v2.0.1 R package (https://cran.r-project.org/web/packages/SKAT) for the analyses, accounting for age, gender, and the top four principal components. No inflation was observed in test statistics as seen in QQ plots (${\lambda }_{{GC}}$ ranged from 0.931 to 0.970; Fig. 1b); population stratification had a negligible effect on the gene-based association tests for rare variants.

#### Rare variants at nAMD-associated GWAS loci

First, we sought to pinpoint rare variants burden at the identified loci for nAMD from our Asian population GWAS by assessing the functional variants in adjacent genes. The *ENTPD4* gene at GWAS locus 8p21.3, from which GWAS lead SNP rs13278062 ($291\,{{{{{{\rm{bp}}}}}}}$ upstream from *TNFRSF10A)* is $204\,{{{{{{\rm{kb}}}}}}}$ downstream (SKAT-O: $\,P=4.49\times {10}^{-4}$ ; SKAT: $P=1.91\times {10}^{-4}$; Table 2 and Supplementary Data 7), was identified as the top gene for the functional variant enrichment for PCV. The *P* value from the kernel SKAT test, but not omnibus test SKAT-O, remained significant after multiple testing corrections for all 190 genes tested at the GWAS loci ($P \, < \, 2.63\times {10}^{-4}$). *ENTPD4*, $\sim 30\,{{{{{{\rm{kb}}}}}}}$ consisting of 14 exons, encodes the apyrase protein, which is an enzyme catalyzing the hydrolysis of nucleotide diphosphates and triphosphates. It comprises 12 missense variants, with mutation counts ranging from 1 to 18. The rare variant burden was not in the shade of LD with the GWAS signals, as suggested from the analysis conditional on the lead GWAS SNP (SKAT test: conditional $P\, < \,4.34\times {10}^{-4}$). Using protein-prediction algorithms (see “Method”), there were 3 rare variants predicted to be damaging (p.L197V, p.P202S, and p.R591H) residing in exons 6 and 13 (Supplementary Data 8). Variant p.P202S exhibited the strongest single-variant association (Supplementary Data 9). *OR4F15* gene at our newly identified GWAS locus 15q26.3 ($\sim 326\,{{{{{\rm{{kb}}}}}}}$ adjacent to GWAS lead SNP *PCSK6* rs7402624) and *LOXL2* gene at GWAS locus 8p21.3 ($\sim 71\,{{{{{\rm{{kb}}}}}}}$ from GWAS lead SNP rs13278062) showed marginal significance for kernel tests. Besides, previously identified rare variants in *FGD6* from WES^26^, which is also a known GWAS locus^13^, exhibited nominal significance (SKAT-O, ${P}=9.41\times {10}^{-3}$; Supplementary Data 7). Single-variant analysis of functional variants for PCV at the GWAS loci identified several rare variants in genes *OR4F15* (p.S20L, $P=1.09\times {10}^{-3}$), and *SELENOS* (p.R54G, $P=2.15\times {10}^{-3}$) at GWAS locus 15q26.3, and two variants in *ENTPD4* (p.P202S, $P=2.54\times {10}^{-3}$; p.S597L, $P=6.32\times {10}^{-3}$; Supplementary Data 9). The significance for the variant p.S20L in *OR4F15* remained at $P=1.56\times {10}^{-3}$, after multiple testing corrections.

**Table 2:** Association of top genes with the rare variant burden for PCV identified from whole-exome gene-based analyses

Gene	Chr	Functional variants (No.)	AA change (minor allele counts)	Gene-based analysis					
				Kernel test	Burden test	SKAT-O test	Kernel test	Burden test	SKAT-O test
Top gene at exome-wide genome							Gene-based analysis on pathogenic variants		
*COL1A1*	17	27	p.A1421T (35), p.S1417Rfs*29 (2), p.E1401K (1), p.A1387S (15), p.V1289L (5), p.A1256T (3), p.R1252S (1), p.E1243K (1), p.S1239N (1), p.A1149S (7), p.V1057I (1), p.R1036C (1), p.M1000I (3), p.R918H (1), c.2452-1G > T (1; splicing), c.2452-2A > C (1; splicing), p.P817A (1), p.A798V (1), p.P739L (1), p.P651S (3), p.Q576H (1), p.S441N (22), p.Q358E (1), p.P333S (1), p.D325G (1), p.M217L (1), p.E77K (1)	$3.57\times {10}^{-7}$	$9.88\times {10}^{-4}$	$1.05\times {10}^{-6}$	$3.14\times {10}^{-7}$	$1.02\times {10}^{-4}$	$8.37\times {10}^{-7}$
Top gene at AMD-associated GWAS loci							Conditional on lead GWAS SNP rs13278062 at GWAS locus 8p21.3		
*ENTPD4*	8	12	p.S597L (13), p.R591H (5), p.V524I (1), p.D521N (4), p.M444V (14), p.Q424R (7), p.T406R (1), p.R396G (10), p.R387Q (7), p.P360L (2), p.P202S (18), p.L197V (1)	$1.91\times {10}^{-4}$	$6.81\times {10}^{-2}$	$4.49\times {10}^{-4}$	$4.34\times {10}^{-4}$	$1.18\times {10}^{-1}$	$9.78\times {10}^{-4}$

259 PCV and 760 controls of Chinese descent were included in the whole-exome sequencing (WES) analyses. Functional variants include missense, stop-gain, stop-loss, frameshifting, or splice-site disruption variants with MAF <2%. Rare variants predicted to be damaging were listed in Supplementary Data 8 and 11.

*AA change* amino-acid change; for two splicing variants, cDNA change indicated, *SKAT-O* sequence kernel association optimal test to combine both kernel and burden tests, *fs frameshifting, *PCV* polypoidal choroidal vasculopathy.

#### *COL1A1* exhibits exome-wide significance for PCV

Across the whole exome, gene *COL1A1* at chromosome 17q21.33 had significant enrichment of rare variants for PCV (SKAT-O, $P=1.05\times {10}^{-6}$; Table 2 and Supplementary Data 10), exceeding the exome-wide gene-level significance at $2.98\times {10}^{-6}$. No signals were seen from the common variants nearby, suggesting that the rare variants’ burden in *COL1A1* was independent of GWAS hits. *COL1A1* encodes the pro-alpha1 chains of type I collagen found in most connective tissues. The majority of 27 functional variants within *COL1A1* were missense variants (24/27), largely residing from exons 15 to 51 (Supplementary Data 11). Ten rare variants were predicted to be damaging, with 8 being singleton variants, indicating the extremely rare functional variants were responsible for the association signals. *ALAS1* at chromosome 3 encoding the mitochondrial enzyme (SKAT-O, $P=2.84\times {10}^{-5}$), and *CCDC175* at chromosome 14 encoding coiled-coil domain-containing protein 175 (SKAT-O, $P=8.10\times {10}^{-5}$) were found to be the top genes at nominal significance. The sensitivity analyses that included variants with MAF $< 1\%$ showed consistent results; the association with *COL1A1* remained the strongest (SKAT-O, $P=1.51\times {10}^{-7}$; Supplementary Data 10), while genes *ALAS1* and *CCDC175* showed nominal significance.

### Heritability and genetic sharing between PCV and typical nAMD

Based on common variants (MAF $> 1\%$), the SNP-heritability of PCV and typical nAMD was estimated at 0.417 (SE $=0.121$) and 0.386 (SE $=0.147$), respectively (Table 3). For the East Asian general population, assuming late AMD prevalence at $0.5\%$^27,28^, the liability-scaled heritability was 0.287 (SE $=0.083$) for PCV and 0.264 (SE $=0.101$) for typical nAMD. The genetic correlation between PCV and typical nAMD was estimated at 0.666 (SE $=0.187$), suggesting substantial sharing of the genetic basis of common variants for these subtypes.

**Table 3:** SNP-heritability estimation for Asian typical nAMD and PCV, and genetic correlation

Phenotype	SNP-*h*^2^				Genetic correlation		
	Observed scale		Liability scale				
	*h*^2^	SE	*h*^2^	SE	*r*_*g*_	SE	*P*
PCV	0.417	0.121	0.287	0.083	0.666	0.187	$4.00\times {10}^{-4}$
Typical nAMD	0.386	0.147	0.264	0.101			

We conducted heritability and genetic correlation analyses using the LD score regression method, applying GWAS summary statistics from 1555 cases with PCV, 1573 cases with typical nAMD and 5493 controls. We retained variants that, after merging with a Hapmap3 SNP list, had a minor allele frequency $$\ge 1\%$$ and an imputation quality info $$ > 0.8$$. We calculated both observed and liability scale SNP-*h*^2^, taking into account the $$0.5\%$$ AMD prevalence in Asian populations^27,28^.

*nAMD* neovascular AMD, *PCV* polypoidal choroidal vasculopathy, *h*^*2*^ heritability estimate, *SE* standard error, *r*_*g*_ genetic correlation estimate.

**Fig. 2:**
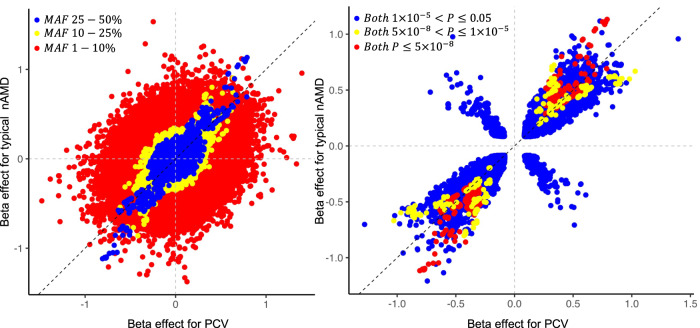
Genetic effect size in PCV versus typical nAMD. For the figure on the left, we grouped variants by minor allele frequency ($1-10\%$, $10-25\%$, or $25-50\%$). Minor allele frequency was computed based on our GWAS data in 8621 subjects of East Asian descent. For the figure on the right, variants were grouped by different association *P* value thresholds ($P\le 5\times {10}^{-8}$, $5\times {10}^{-8}\, < \,P\le 1\times {10}^{-5}$, or $1\times {10}^{-5}\, < \,P\le 0.05$) in GWAS for both PCV and typical nAMD. We conducted a single-variant test using the firth bias-corrected likelihood-ratio model, incorporating the top four principal components as covariates. ${\chi }^{2}$ statistics with one degree-of-freedom were used for the single-variant test. We then performed a meta-analysis under an inverse-weighted fixed-effect model, combining data from all cohorts for each phenotype. Each data point represents a variant out of the total 7,911,145 overlapped variants in PCV and typical nAMD GWAS. The black diagonal dashed line represents the same effect size between PCV and typical nAMD. The gray horizontal and vertical dashed lines at the value of 0 separate the figure into four quadrants. Variants in the first and the third quadrants have a consistent direction of effect between the two AMD subtypes.

To evaluate whether genetic effect sizes were consistent at varying degrees of MAF, we compared effect sizes for each subtype at different MAF ranges and *P* values (Fig. 2). We grouped these variants by allele frequencies ($1-10\%$, $10-25\%$, and $25-50\%$) and *P* values ($P\le 0.05$, $P\le 1\times {10}^{-5}$ and $P\le 5\times {10}^{-8}$). The effect sizes and direction were largely consistent for common or high-frequency variants but exhibited variations to some extent for low-frequency variants. To identify those exhibiting the largest difference in the effects, we performed GWAS for PCV vs. typical nAMD. The QQ plot indicated a genomic inflation factor ${\lambda }_{{GC}}$ at 0.957, with no evidence of potential population substructures (Supplementary Fig. 6). The *ARMS2* rs61871744 variant remained the most significant signal (${P}_{{{{{{{\rm{dif}}}}}}}}=1.91\times {10}^{-8}$; Supplementary Data 12 and Supplementary Fig. 7), corroborating previous findings^29^. Thirteen loci showed marginal significance at *P* value less than $1\times {10}^{-5}$, with opposite genetic effects observed for each subtype. However, none of these loci showed genome-wide significance in subtype-specific analysis.

Supplementary Data 13 summarizes the implicated genes for AMD, PCV, and typical nAMD, from previous large-scale genome studies^12–20,22,24–26^. along with ours. A large body of AMD genes is discovered in European samples; the most significant genes showing the strongest effect size for European advanced AMD are shared in Asian nAMD. Novel genes identified for PCV are mainly from Asian GWAS or WES.

### Fibrosis and collagen-related pathways for PCV

We further conducted pathway analysis on 15,481 gene sets. After correction for multiple testing, fibroblast senescence, which elevates fibroblast numbers leading to tissue fibrosis and stiffening in the elderly^30^, and collagen extracellular matrix (ECM) assembly, were identified as top pathways for PCV (LY_AGING_OLD_UP, M8910, $P=2.28\times {10}^{-7}$; go_collagen_type_iv_trimer, M25627, $P=1.61\times {10}^{-5}$, respectively; Supplementary Data 14). *HTRA1*, along with several genes (e.g., *CST6, FMOD, COMP*) are involved in fibroblast senescence. The collagen ECM assembly pathway requires type IV collagen triple helices to form three-dimensional networks within the basement membrane and involves *COL4A4* and *COL4A3* genes. The corresponding genes involved in the key pathways associated with PCV are listed in Supplementary Data 15. These two pathways, in contrast, had much weaker signals for typical nAMD ($P=0.017$ and 0.032 respectively).

### eQTL analyses identify multiple potential target genes at the novel loci

To identify potential genes at the four novel loci—*GATA5/LAMA5*, *PCSK6, SPATA13* and *NEK6/LHX2*, we performed GWAS SNP expression quantitative trait locus (eQTL) association using GTEx data across 53 human tissues. *GATA5* was the most significant novel locus identified in our study. Within the *GATA5* locus, comprised of about 30 genes in a $500\,{{{{{\rm{{kb}}}}}}}$ region, SNP rs6121609 was associated with the expression of eQTL genes including *GATA5*, *LAMA5* and *MTG2* ($P\, < \,9.43\times {10}^{-4}$; Supplementary Data 16). *CABLES2* showed nominal association at $P=9.44\times {10}^{-4}$. Several other genes (e.g., *RBBP8NL, TCFL5* and *COL9A3*) showed marginal significance ($P\, < \,5\times {10}^{-3}$); among these, *MTG2*, *TCFL5* and *COL9A3* were about $100\,{{{{{\rm{{kb}}}}}}}$ away from the top peak. For the other 3 loci, the genes where the top hits reside, or the nearest genes, were found to be the top eQTL genes (e.g., *NEK6/LHX2*, eQTL gene: *NEK6*; *SPATA13*, eQTL gene*: SPATA13*; $P\, < \,9.43\times {10}^{-4}$).

### Functional characterization of target genes at *GATA5* locus and of *COL1A1* gene

We employed preclinical models of ocular angiogenesis and subretinal fibrosis to examine the gene expression of potential eQTL genes identified at the *GATA5* locus and *COL1A1* gene identified by WES. Genes that showed low expression at the RNA level, or no expression at the protein level in eyes in the Human Protein Atlas database (HPA; https://www.proteinatlas.org/), were omitted in further analyses. Four genes—*LAMA5, MTG2, CABLES2, and COL9A3*—along with *COL1A1*, were included for functional studies. *GATA5*, the nearest gene adjacent to the lead variants, had low expression in the neuroretina and RPE/choroid complex in normal mice (threshold cycle value $$ > 30$$). We thus examined the expression of *GATA5* only in the mouse model of laser-induced choroidal neovascularization and human RPE cells for retinoic acid treatment.

#### mRNA expression patterns in vivo ocular angiogenesis models

**Fig. 3:**
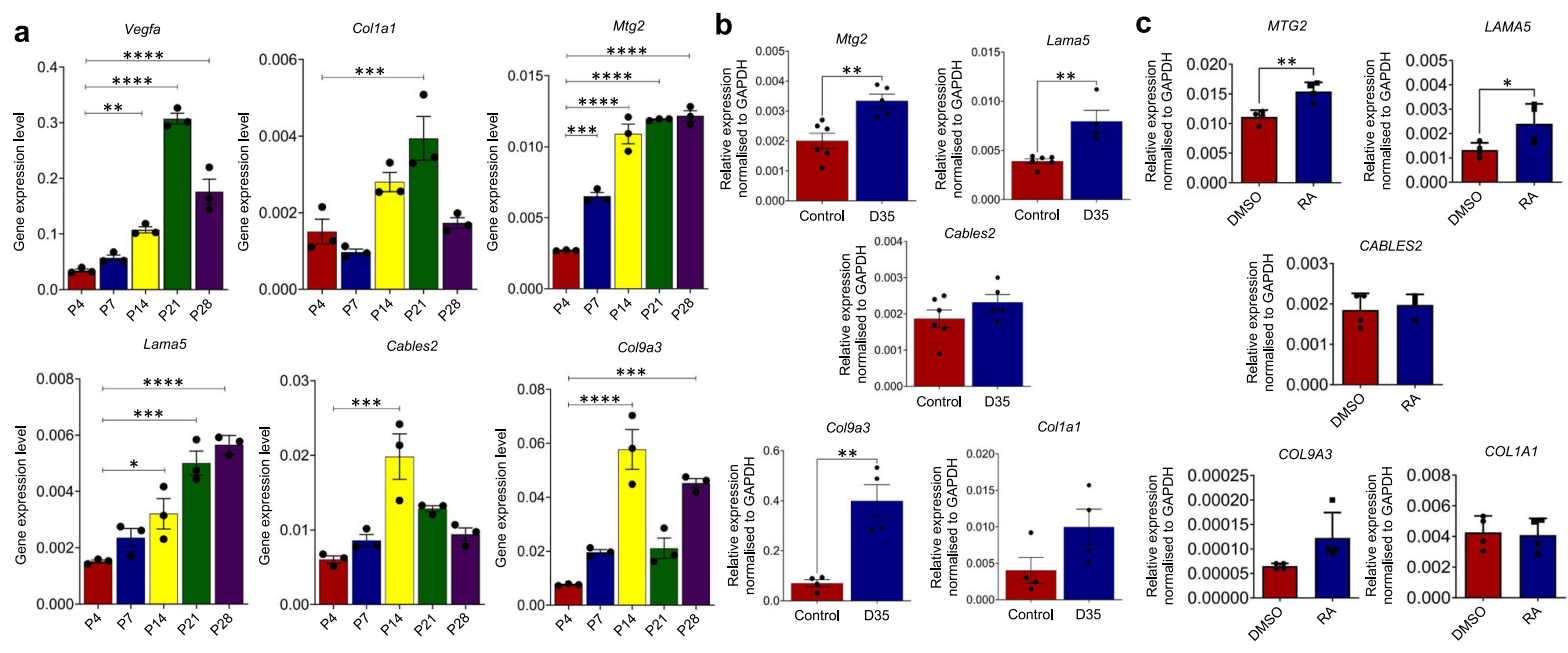
Comparative mRNA expression analysis of selected genes in the retinal tissue of developing mice, RPE cells following the laser-induced CNV in mice, and human retinal pigment epithelial (HRPE) cells upon retinoic acid treatment. **a** mRNA expression of *COL1A1* and gene candidates at *GATA5* locus in the retinal tissue of developing mice. Each group included three mice as test subjects. The expression profiles of selected gene targets from the mouse retina were analyzed on postnatal days P4, P7, P14, P21, and P28, as shown on the *y*-axis of bar plots ($$n=3$$ animals). Significant differences in gene expression level were observed between P4 and P14, P21, and P28 for *Vegfa* (*P* values: 0.0041, $$ < 0.0001$$, and $$ < 0.0001$$, respectively). For *Col1a1*, the *P* value is $$ < 0.001$$ for P4 vs. P21. For *Mtg2*, significant differences were found for P4 vs. *P*7, P14, P21, and P28 (*P* value are $$0.0001$$, $$ < 0.0001$$, $$ < 0.0001$$, and $$ < 0.0001$$, separately). For *Lama5*, significant differences were observed for P4 vs. P14 (*P* value is $$0.0215$$), P4 vs. P21 (*P* value is $$0.0001$$), and P4 vs. P28 (*P* value $$ < 0.0001$$). For *Cables2*, the *P* value is $$0.0005$$ for P4 vs. P14. For *Col9a3*, significant differences were observed for P4 vs. P14 and P4 vs. P28 with *P* values $$ < 0.0001$$ and $$=0.0001$$, respectively. **b** Target genes induced in RPE cells following the laser-induced CNV in mice. qRT-PCR analysis of each gene in RPE cells isolated from mice at day 35 following laser-induced CNV. For *Col1a1* and *Col9a3*, experimental groups had four mice each. *Lama5*’s control group had six mice, and D35 group had four mice. For *Cables2* and *Mtg2*, the control group had six mice, and the D35 group had five mice. The *P* values for *Mtg2*, *Lama5*, *Col9a3*, *Col1a1*, and *Cables2* are $$0.004$$, $$0.003$$, $$0.003$$, $$0.095$$, and $$0.203$$, respectively. **c** mRNA expression of gene candidates in human retinal pigment epithelial (HRPE) cells upon retinoic acid treatment, compared to induction using dimethyl sulfoxide (DMSO) solvent in four independent experiments. *P* values for gene candidates are: *MTG2* ($$0.016$$), *LAMA5* ($$0.048$$), *COL9A3* ($$0.070$$), *COL1A1* ($$0.854$$), and *CABLES2* ($$0.627$$). For all independent experiments, the mRNA expressions of lead gene candidates were measured using qRT-PCR method. Source data are provided as a Source Data file. Error bars were presented as mean $$\pm$$ S.E.M. Statistical significance was determined by one-way ANOVA or two-tailed, unpaired Student’s *t* test, $$*P\, < \,0.05$$, $$*\ast P\, < \,0.01$$, $$*\ast*P\, < \,0.001$$, and $$*\ast*\ast P\, < \,0.0001$$.

The murine retina provides an ideal model for studying sprouting angiogenesis^30^. To establish the association between lead gene candidates and developmental angiogenesis in the eye, the expression profiles of selected gene targets were analyzed in the neuroretina of mice at postnatal days P4, P7, P14, P21, and P28 (Fig. 3a). The superficial vascular network of the retina formed at P4 in our study suggests that all candidate genes were expressed at low levels in the P4 retinae. At P7, the superficial vascular plexus began to mature and branch vertically to reach the deep vascular plexus during the second week after birth^31^. At this stage, the vascular network fully matured throughout the retina. At P14, the intermediate vascular plexus began to form in the retinae, and at P21, the plexus developed deeper and the hyaloid vessels regressed. The maturation of the retinal vessels, especially the intermediate plexus, continued until P28. The expression of *Col1a1* in the retinae increased gradually after birth and reached its highest levels at P21 (2.6-fold increase compared to that at P4, $P\, < \, 0.001$) which mirrors the expression pattern of *Vegfa*, a master regulator of angiogenesis vascular endothelial growth factor (9-fold increase, $P \, < \, 1\times {10}^{-4}$). On the contrary, *Mtg2* and *Lama5* expressions continued to increase till P28, suggesting that these two genes may also be involved in retinal vessel remodeling (4.5-fold increase, $P \, < \, 1\times {10}^{-4}$; 3.7-fold increase, $P=1\times {10}^{-4}$, respectively). The expression levels of *Cables2* and *Col9a3*, on the other hand, peaked at P14 when retinal angiogenesis reached its highest level (3.3-fold higher than those at P4, $P=5\times {10}^{-4}$; 7.6-fold higher, $P \, < \,1\times {10}^{-4}$, respectively), significantly decreasing thereafter at P21.

To understand the roles of the above genes in the development of choroidal vasculature, we further examined the expression levels in the mice RPE/Choroid complex at postnatal days P4, P14, and P28 respectively (Supplementary Fig. 8). Similar to *Vegfa*, *Lama5* expression was higher at P14 compared to that at P4 ($P=0.021$). Other genes showed lower (*Cables2* and *Col9a3*) or similar expression levels (*Mtg2*) at P14 and P28, whereas *Col1a1* level was down-regulated at P28.

#### Induction of *Lama5*, *Mtg2*, and *Col9a3* expression in vivo subretinal fibrosis models

The mouse model of laser-induced choroidal neovascularization (CNV) serves as a good model to study subretinal fibrosis^32^, a characteristic feature of patients with advanced nAMD. The extent of subretinal fibrosis becomes apparent at day 35 post-laser treatment^33^. Retinal Pigment Epithelium (RPE) cells are key contributors to the abnormal accumulation of extracellular matrix (ECM) components in the subretinal space of nAMD patients^34^. To evaluate the association between novel target genes and subretinal fibrosis, the expression of gene candidates in the RPE/choroid tissues isolated from C57BL/6 mice at day 35 following the induction of CNV by laser photocoagulation was analyzed by qRT-PCR. Our study showed the following expression pattern in CNV eyes with subretinal fibrosis compared to those in non-lasered controls: 2-fold increase in *Lama5 (*$P=0.003$), 1.7-fold increase in *Mtg2* ($P=0.004$) and 5.7-fold increase in *Col9a3* ($P=0.003$), and no significant change in *Cables2* (1.2-fold, $P=0.203$) in (Fig. 3b); *Col1a1* expression level was induced 2.5-fold, but the increase was not statistically significant ($P=0.095$). For *Gata5*, the expression level was low and there was no significant difference in *Gata5* expression in the RPE/choroid tissues at day 35 post-laser treatment (Supplementary Fig. 9A).

#### Retinoic acid induces *LAMA5* and *MTG2* expression in Human RPE cells

Our in silico analysis showed that the top 30 SNPs at the *GATA5* locus reside in the putative transcription factor binding sites (TFBS; Supplementary Fig. 10). Intriguingly, the major transcription factors (TFs) are responsive to retinoic acid and glucocorticoid receptor signaling. To establish the association between RA and the above gene candidates expressed in the eye, human retinal pigment epithelial (HRPE) cells were treated with retinoic acid, and the mRNA expression of each gene was measured using qRT-PCR. Our study showed that 24-h treatment with 10 μm retinoic acid had no obvious impact on RPE cell morphology and viability (Supplementary Fig. 11). Both *LAMA5* and *MTG2* were significantly induced by retinoic acid in RPE cells (1.8 fold, $P=0.048$; 1.4 fold, $P=0.016$ respectively; Fig. 3c). On the other hand, dexamethasone could not induce the expression of any candidate genes in HRPE cells (Supplementary Fig. 12). For *GATA5*, the expression level was low in HRPE cells, and RA treatment had no impact on the *GATA5* expression (Supplementary Fig. 9B). Our data suggest that retinoic acid-responsive genes (e.g., *LAMA5* and *MTG2*) may be involved in the pathophysiology of nAMD. Further investigations are needed to confirm this hypothesis.

## Discussion

The current study is the most comprehensive meta-analysis of GWAS for Asian nAMD and PCV in East Asian populations, at both common and rare variants. We identified four novel loci (the strongest association at *GATA5* locus) for Asian nAMD, and rare variant mutations enriched gene (*COL1A1*) for PCV. Using human multi-tissue eQTL analysis and the murine model, we prioritized potential target genes, including *LAMA5, MTG2* and *COL9A3* at the *GATA5* locus. These genes were induced in mouse eyes with ocular angiogenesis and subretinal fibrosis, characteristic pathological features of nAMD. We further showed retinoic acid, critical for visual pigment recycling, increased the expression of *LAMA5* and *MTG2* in HRPE cells. While PCV and typical nAMD exhibited substantially high genetic correlation and shared top variants, collagen ECM assembly and fibrosis-related pathways were more pronounced for PCV.

Our study highlights the role of collagen-related genes associated with PCV. We found genome-wide significance at the *COL4A4*, and using the exome-sequencing approach, we identified functional mutations in the *COL1A1* gene that are associated with PCV. Protein-altering variants result in altered residual changes in the collagen alpha 1 chain triple helical domain and C-terminal propeptide (COLFI)^35^; the latter controls procollagen intracellular assembly and the extracellular assembly of collagen fibrils. The rare mutations identified in *COL1A1* have not been previously reported as ClinVar, except p. A1256T for several Mendelian diseases, such as Ehlers-Danlos syndrome^36^, a connective tissue disorder that is characterized by joint hypermobility, tissue fragility, and skin abnormalities. *COL1A1* has also been reported as a keratoconus-associated gene^37^. In the eye, COL1A1 represents one of the prominent ECM components driving subretinal fibrosis^38–40^; mutations in type I collagen have been reported to cause dysregulated TGFβ signaling^41^, a pathway that chiefly regulates tissue fibrosis by controlling TGFβ bioavailability. Intriguingly, pathway analysis based on common variants revealed that fibrosis and collagen ECM pathways are more pronounced for PCV, with the involvement of type 4 collagen genes (e.g., *COL4A4*). Furthermore, the non-collagenous (NC) domain of various collagens has been implicated in angiogenesis^42,43^. For example, the NC1 domain of α1, α2, α3, and α6 chains of collagen IV could be released via proteolytic cleavage, which inhibited angiogenesis^44–46^. However, an antiangiogenic role for *Col1a1* or the protein it encodes has not been established to date. It will be interesting to study how PCV-associated functional mutations in *COL1A1* and *COL4A4* affect ECM rigidity, as well as fibrosis and angiogenesis pathways in the future.

Among the four novel loci identified for nAMD, *GATA5* has emerged as an interesting AMD-associated locus. GWAS carried out on the Japanese population (${r}^{2}=0.931$)^47^ showed that rs6121609, the top SNP identified, is in LD with rs6061548, which is known to be associated with central serous chorioretinopathy (CSC). Both minor alleles were associated with a higher risk of AMD and CSC, in contrast to the opposite effect observed at *CFH* rs800292 for AMD and CSC^48^. Overall, our data provide further genetic evidence for the link between AMD and CSC.

It is challenging to assign the non-coding GWAS associations at GWAS loci to causal variants or genes. We fine-mapped the functional rare variants using exome sequencing data on genes identified at GWAS loci. We discovered the enrichment of rare mutations in the *ENTPD4* gene for PCV at the GWAS locus 8p21.3. *ENTPD4* encodes NTPDase4, a member of the apyrase protein family which belongs to the same family as CD39^49^, the latter tightly regulating ATP signaling as NTPDase^50^. High levels of extracellular ATP released by stressed cells can cause cytotoxic calcium influx^51–53^ and increase the vulnerability of RPE cells^54,55^. *ENTPD4* is highly expressed in endothelial, neuronal and glial cells of the retina^56,57^. We speculate that NTPDase4-regulated ATP signaling has a potential role in contributing to unique vascular and RPE cell pathologies in PCV patients. The majority of genes harbored in GWAS loci, including the *GATA5* locus, did not exhibit significant functional rare variant enrichment; this is in part due to moderate effects of the rare variants, or distal target genes responsible for the signal.

We further characterized the lead SNPs located at ~$230\,{{{{{\rm{{bp}}}}}}}$ from the 3’ end of *GATA5* gene. Our in silico analysis showed that the majority of variants reside in the putative binding sites of transcription factors that are responsive to retinoic acid and glucocorticoid receptor (GR) signalings. We have shown that the expression of *LAMA5* and *MTG2* were significantly induced by retinoic acid in human RPE cells; this is concordant with our in silico analysis, suggesting that retinoic acid signaling may serve as an important mechanism of SNP association at the *GATA5* locus in nAMD patients. Both in vitro and in vivo studies have shown all-trans-retinol acid to be a pro-angiogenic factor for nAMD^58^, which directly transactivates angiogenic factors such as VEGF in RPE cells via retinoic acid receptors/retinoid X receptors binding sites in their prompter regions^59^. *MTG2* and collagens were also previously shown to be regulated by retinoic acid in non-ocular tissues^60^. Although endothelial GATA5 was previously reported to regulate angiogenesis^61^, it is expressed at low levels in human and mouse ocular tissues as well as human RPE cells. In addition, *GATA5* expression was not induced in the RPE/choroid complex of mouse eyes subjected to laser-induced choroidal neovascularization and fibrosis. Our data do not support the involvement of *GATA5* in AMD pathologies. Further studies are needed to establish the direct association between lead SNPs at the *GATA5* locus and the expression of target genes in ocular cells, identify the role of retinoic acid-responsive transcription factors in regulating the expression of target genes, and explore the functional consequences of altered gene expression on AMD pathologies.

Furthermore, the subretinal fibrosis models in our study showed higher expression levels of the target genes at the *GATA5* locus including *LAMA5*, *MTG2*, and *COL9A3*. Clinically, fibrosis is known to be an important predictor of vision outcomes, following anti-VEGF treatment^62–64^. Interestingly, our study has suggested that the prevalence of fibrosis increased 3 folds from $13.0$ to $37.8\%$ between baseline and 12 months of AMD diagnosis in Singapore patients^64^. The prevalence is much higher than in the European sample. Further characterization of AMD genes responsible for fibrosis might shed light on alternative treatment strategies beyond anti-VEGF.

Besides the *GATA5* locus, three novel loci were identified including *NEK6/LHX2, SPAT13* and *PCSK6*. *NEK6/LHX2* locus was associated with typical nAMD, but exhibited a much weaker signal for PCV. *NEK6* expression was induced by hypoxia in a Hif1a-dependent manner^65^ and involved in key angiogenic pathways^66^. LHX2 has been reported to activate mTOR and PDGF signaling, pathways associated with nAMD^67,68^. Both genes are highly expressed in muller glial cells of the eye^56^. Muller cells lose the linear shape and become migratory in typical nAMD and develop into a membrane in the region of the retina with the presence of CNV^69,70^. However, little attention has been given to muller cell changes in eyes with PCV. The NEK6/LHX2-mediated changes in muller glial cells might underlie the different pathogenesis of PCV and typical nAMD. For *SPATA13* gene, it has been reported to be associated with primary angle-closure glaucoma pathogenesis, by regulating nucleotide exchange factors activity and affecting homeostasis in the mitosis in the retina, RPE, cornea and lens etc.^71^. For *PCSK6*, it encodes the proprotein convertase subtilisin/kexin family; the key function of *PCSK6* has been reported to be involved in lipid metabolism, atherosclerotic plaques, ECM remodeling and cytokines^72^. How these genes-mediated changes in the eye underlie the different pathogenesis of nAMD warrants further investigation.

Although the underlying genetic effects of common variants were largely shared between PCV and typical nAMD, certain variants exhibited some degree of variation in the genetic effects. Through exome sequencing approaches, we identified rare variant burden for PCV (e.g., *COL1A1*), and replicated the previously reported gene *FGD6*^26^. For common variants, although largely concordant, some subtle differences were observed between subtypes. For example, PCV patients have different genetic effect sizes at the same index SNPs (i.e., *ARMS2/HTRA1, NEK6/LHX2*), or manifested the association at independent variants (i.e., *CO14A4/COL4A3*). Taken together, our study highlights the potential of employing common and rare variants identified through whole-genome scans to determine variants or genes for PCV in East Asians.

In summary, we have identified novel loci for nAMD and PCV in East Asians, through both common and rare variant association analyses. Additional functional studies have identified potential candidate target genes at the *GATA5* locus for nAMD. Our findings shed light on the underlying molecular mechanisms of nAMD in Asian populations, providing insights for the development of novel Asian-specific treatment strategies.

## Methods

All studies were performed with the approval of their Human Research and Ethics Committee, adhering to the Declaration of Helsinki principles. Written informed consent was obtained by the ethics committee of all the participating institutions, which include Singapore National Eye Center, National University Health System, Tan Tock Seng Hospital, Hong Kong Eye Hospital, Prince of Wales Hospital Eye Center, Sun Yat-sen University Cancer Center, Department of Ophthalmology at Kyoto University Hospital, Fukushima Medical University Hospital, Kobe City Medical Center General Hospital, Ozaki Eye Hospital, Mizoguchi Eye Clinic, Japanese Red Cross Otsu Hospital, Nagahama City Hospital, Seoul National University Bundang Hospital, Seoul National University Hospital, Kyungpook National University Hospital, Yeungnam University Hospital, Kosin University Hospital, and Busan Paik Hospital.

### Study samples and phenotyping

We performed GWAS and WES analyses for investigating nAMD associations in East Asians from the GAMA Consortium. The GAMA consortium includes data from four case-control studies from Singapore, Hong Kong, Korea and Japan, each of which contains: (1) cases with polypoidal choroidal vasculopathy (PCV), (2) cases with typical nAMD, and (3) hospital-based controls without any clinical signs of AMD, or general controls enrolled from population-based studies. Cases with other macular diseases such as central serous chorioretinopathy, high myopia, and angioid streaks were excluded. Details of the sample recruitment procedure are provided in Supplementary Notes. A total of 3128 cases (1555 PCV patients and 1573 typical nAMD patients) and 5493 controls were included in the analyses, and for the WES analysis, 1019 Chinese subjects (259 PCV and 760 controls) were recruited from Singapore (Supplementary Fig. 1). For all studies, the diagnosis of advanced AMD phenotyping followed the Age-Related Eye Disease Study (AREDS) or Wisconsin age-related maculopathy grading system based on dilated fundus photography (Supplementary Notes). For the presence of choroidal neovascularization, grading of fluorescein angiography was performed using a modification from the Macular Photocoagulation Study^73^. Indocyanine green angiography was performed to diagnose PCV using the Japanese Study Group guidelines^74^.

### Genotyping, quality control, and imputation

The DNA samples were genotyped on Illumina OmniExpress-12 and Human610-Quad Bead Chips. Details of genotyping platform, number of variants and phenotyping are provided in Supplementary Data 1.

The centralized quality control (QC) procedure for GWAS genotyping data was applied to each study. For variant-based quality control, genetic variants with minor allele frequency (MAF) $$ < 0.5\%$$, genotype call rates $$ < 95\%$$, or deviation from Hardy-Weinberg equilibrium (HWE) ($$P\, < \,1\times {10}^{-6}$$) were excluded. Here, we only included autosomal variants in the analysis. For sample-based quality control, participants with relatedness corresponding to halfway of second- and third-degree relatives or closer (Identity-by-decent [IBD] $$ > 0.1875$$), as well as samples with missing genotype calls $$ > 5\%$$ or an excess of heterozygosity ($$ > 6$$ SD) were excluded. For related samples in family clustering, one individual with the lowest missing rate of genotypes was retained. The analyses above were performed using PLINK 1.90 beta (https://www.cog-genomics.org/plink/) and R 3.5.3 (https://cloud.r-project.org/). Principal component analysis (PCA) was conducted to detect outlier samples using EIGENSOFT v7.2.1 (https://github.com/DReichLab/EIG/archive/v7.2.1.tar.gz). Reference data were based on HapMap 3 of Japanese, Han Chinese, Europeans, Yoruba, and Mexican populations and SEED data of Indian and Malay populations^75^. Prior to performing imputation, we excluded variants that were not present in 1000 Genomes Phase 3 of the East Asian population or had mismatched chromosomes, base pairs, or alleles using the HRC script. We removed palindromic variants (A/T or G/C) with MAF $$ > 0.4$$ and variants with an allele frequency difference $$ > 0.2$$ in our data and the 1000 Genomes Phase 3 reference panel of the East Asian population. All variants were aligned to the forward strand, and reference alleles were fixed according to the reference panel.

Genotyping was conducted using Illumina OmniExpress-12 v1 on 714,255 variants in the Japan study. After applying QC procedures, we retained 2113 Japanese participants (956 cases and 1157 controls) and 579,490 variants. In the Korean cohort, we utilized the same Illumina OmniExpress-12 v1 for genotyping and, after QC, retained 574,860 variants in 1384 participants (485 cases and 899 controls). For the Hong Kong study, we performed genotyping for Chinese participants using Illumina OmniExpress-12 v1 in cases on 714,255 variants and Human610-Quad BeadChip in controls on 573,871 variants. After applying QC, we included a total of 295,943 variants common for all samples (466 cases and 1006 controls) which was used for imputation.

For the Singapore study, the genotyping was performed in Chinese participants using Human610-Quad BeadChip v1 or Illumina OmniExpress-12 v1 on up to 714,255 variants. We conducted QC at the batch level because genotyping was done in subsequent batches for cases. QC was done in the following steps: (1) at each batch, variant-based quality control was employed using the same criteria as above. We ensured a forward strand for each batch based on 1000 Genomes Phase 3 reference panel of the East Asian population. (2) We conducted a Fisher exact test on the genotype counts for batch effects for cases. We tested the null hypothesis that a given batch has the same genotype frequencies as other batches combined. Variants with a *P* value less than $1\times {10}^{-6}$ were excluded in all batches. (3) We combined all batches and re-performed variant-based quality control with the same criteria as in the first step. (4) Sample-based quality control was then performed as above. Finally, 283,411 post-QC variants common for 3652 Chinese participants (1221 cases and 2431 controls) were used for imputation.

We imputed whole-genome variants for each study separately based on the post-QCed genotypes, with the 1000 Genomes Phase 3 mixed populations as reference panel, using Minimac4 on the Michigan Imputation Server (https://imputationserver.sph.umich.edu/index.html#!pages/home). For the Hong Kong and Singaporean cohorts, we used the intersection of genotyped SNPs from different genotyping arrays in cases and controls for imputation^76^.

### Genome-wide single-variant association analysis and meta-analysis

We conducted a single-variant test using the firth bias-corrected likelihood-ratio model implemented in EPACTS v3.3.0 (https://genome.sph.umich.edu/wiki/EPACTS). For each cohort, we performed case-control association tests under an additive model for each genotyped or imputed variant with all nAMD, PCV, and typical nAMD, separately. The number of risk alleles is an integer (0, 1, and 2) for directly genotyped SNPs, or allele dosage probability ranging from 0 to 2 for imputed SNPs. The top four principal components were included as covariates to account for the population substructure. We performed a meta-analysis under an inverse-weighted fixed-effects model across all cohorts for each phenotype, and filtered out variants with imputation quality $$ < 0.5$$. Genetic variants of imputation quality $$\ge 0.5$$ in at least 3 studies were included in the meta-analysis. Inter-study heterogeneity was reported based on heterogeneity statistic $${I}^{2}$$. *P* value of $$5\times {10}^{-8}$$ was considered as genome-wide significance. We also perform a random-effect meta-analysis using GWAMA v2.2.2^77^ (https://www.geenivaramu.ee/tools/GWAMA_v2.2.2.zip), which estimated heterogeneity variance with the DerSimonian-Laird method.

To identify independently-associated variants, we performed the clumping analysis using FUMA v1.3.6a^31^ (https://fuma.ctglab.nl/). The significant variants were identified ($P\, < \,5\times {10}^{-8}$) in each clump, which was formed of all other variants that were within $500\,{{{{{\rm{{kb}}}}}}}$ from the index variant (${r}^{2} > 0.050$). The variant with the smallest associated *P* value in each clump was considered the independent lead variant. A locus was identified by the independent lead variant with the regions flanking $500\,{{{{{\rm{{kb}}}}}}}$ on both sides. Single-variant annotation was performed using ANNOVAR (https://annovar.openbioinformatics.org/en/latest/).

Conditional analysis was performed using GCTA 1.92.4 beta version (https://cnsgenomics.com/software/gcta/#Download)^78^. Adjusting for the lead variant, the variants in a 1 Mb region at each locus were extracted for the association analyses. Additional independent variants were defined as $P\le 5\times {10}^{-8}$ in original GWAS and conditional analysis. The LD reference data was the original GWAS data.

Furthermore, we also conducted GWAS by using 1555 PCV patients as cases and 1573 typical nAMD patients as controls. A single-variant firth regression was performed using EPACTS v3.3.0.

### Whole-genome gene-based and pathway analysis

The gene-based analysis tested the whole-genome variant associations using the meta-analyzed GWAS summary statistics. The variants were annotated to genes based on the base pairs, adopting a $10\,{{{{{\rm{{kb}}}}}}}$ window at both sides. Analyses were performed using MAGMA^79^ as implemented in FUMA v1.3.6a^80^. The major histocompatibility complex (MHC) region was omitted from the analysis. A total of 18,830 protein-coding genes based on Ensemble v92 were included. A threshold *P* value of $0.05/18,830=2.66\times {10}^{-6}$ was used to demonstrate statistical significance, after correcting for multiple testing using the Bonferroni method. For pathway analysis, gene-based *P* values were used to test 5497 curated gene sets (including canonical pathways) and 9984 GO terms from MsigDB v7.0^81^. A threshold *P* value of $0.05/{{{{{\mathrm{15}}}}}},{{{{{\mathrm{481}}}}}}=3.23\times {10}^{-6}$, derived after Bonferroni correction, was considered statistically significant in the pathway analysis,

### Processing and QC of whole-exome sequencing data

Whole-exome sequencing was done for 1098 samples (301 cases and 797 controls) using the Nimblegen SeqCap EZ (Roche) exome kit. Samples were sequenced to high coverage (target $50\times$) on the Illumina HiSeq 1000 platform or NovaSeq 6000 using $150\,{{{{{\rm{{bp}}}}}}}$ or $100\,{{{{{\rm{{bp}}}}}}}$ paired-end sequencing.

For read mapping and variant analysis, the resulting reads were aligned to a human reference genome (GRCh37) using Burrows-Wheeler Aligner (BWA, v0.7.10; https://sourceforge.net/projects/bio-bwa/files/)^82^. The adapters and sites with a low Phred score ($$ < 20$$) were filtered and data were processed with the Genome Analysis ToolKit (GATK v4.0.3.0) best practices workflow to obtain jointly genotyped VCF files^83^. Reads were locally realigned (GATK IndelRealigner), and their base qualities were recalibrated (BQSR; GATK Recalibration Table). We performed multi-sample joint calling to generate single-nucleotide variant (SNV) calls across all samples^84^. Genotypes were first filtered by the GATK variant quality score recalibration (VQSR). Additional filters excluded the sites with low depth rates ($$ < 10\times$$), high depth rates ($$ > 300\times$$), excessive heterozygosity ($$P\, < \,1\times {10}^{-6}$$), HWE *P* value $$ < 1\times {10}^{-4}$$, or missingness rate $$ > 10\%$$. The media coverage of the target was $$32\times -52\times$$. (Supplementary Fig. 12).

For data quality control, we excluded 37 controls with early AMD, three typical nAMD patients and one Malay case. The rest of the 1057 individuals were of Chinese ancestry based on the PCA results. PCA was performed by GCTA v1.92.4 beta. We further excluded 38 cases with a missingness rate of genotypes $> 10\%$. After variant QC, we included 637,799 variants for 1019 individuals (259 PCV and 760 controls). Our data included two batches of cases (137 and 122 subjects) derived using $150\,{{{{{\rm{{bp}}}}}}}$ or $100\,{{{{{\rm{{bp}}}}}}}$ paired-end sequencing. Thus, we performed a Fisher exact test using genotype calls to check the batch effect. Seventeen markers that failed to pass the *P* value threshold of $1\times {10}^{-6}$ were set to missing. Among the remaining 637,782 variants, 159,036 were exonic nonsynonymous, and 43,918 had a minor allele frequency (MAF) $> 0.1\%$. Most of the detected SNPs were rare variants: $83.41\%$ of them were found with MAF $< 1\%$, and $88.50\%$ were found with MAF $< 5\%$.

### Association analysis of rare variants

We performed both single-variant and gene-based analyses to test the association of rare variants’ burden on PCV. For single-variant analysis, a logistic regression model with the Firth correction was performed using EPACTS v3.3.0, adjusted for age, gender, and the top four principal components. Those variants with MAF $$ > 0.1\%$$ (or equivalently minor allele count [MAC] $$ > 2$$) were included in the single-variant analysis. For gene-based analysis, we applied the Burden test, SNP-set (Sequence) Kernel Association Test (SKAT), and SNP-set Kernel Association Optimal Test (SKAT-O) on variants with MAF $$ < 2\%$$. In total, 145,829 variants used for the test were assigned to 16,794 genes. Analyses were implemented in an R package “SKAT” v2.0.1 (https://cran.r-project.org/web/packages/SKAT). The *P* value threshold was set at $$2.98\times {10}^{-6}=0.05/{{{{\mathrm{16,794}}}}}$$, to be considered statistically significant in the gene-based analysis. In addition, we conducted a sensitivity analysis on rare variants at MAF $$ < 1\%$$. A total of 142,783 variants were mapped to 16,765 genes, with the *P* value threshold setting at $$0.05/16,765=2.98\times {10}^{-6}$$.

### Functional variants annotation

We annotated the variants which passed QC using ANNOVAR. The functional variants that alter peptide sequences (missense), or lead to stop-gain, stop-loss, frameshifting, or splice-site disruption were included in the association analysis. Functional effects of variants were predicted by three different algorithms: SIFT^85^, PolyPhen-2^86^, and Combined Annotation Dependent Depletion (CADD)^87^. Missense variants that had met the following criteria were computationally predicted to be damaging or deleterious: (1) probably damaging (D) in PolyPhen; (2) deleterious in SIFT (D) and (3) CADD $$ > 20$$. CADD C-score greater than 20 indicates variants ranking in the top $$1\%$$ of most deleterious mutations in the human genome^87^.

### Heritability and genetic correlation

We assessed the SNP-heritability (SNP-*h*^2^), as well as the genetic correlation between PCV and typical nAMD using LD score regression (LDSC v1.0.1; https://github.com/bulik/ldsc), based on 888,842 Hapmap3 common variants with MAF $$\ge 1\%$$ and imputation quality info $$ > 0.8$$. The 1000 Genomes Phase 3 reference panel of the East Asian population was used to compute linkage disequilibrium scores. We reported SNP-*h*^2^ and genetic correlation at the liability scale to account for AMD prevalence, assuming the prevalence of AMD is $$0.5\%$$ in the East Asian general population^27,28^.

### Expression quantitative trait locus (eQTL) analysis

We conducted eQTL analysis for the non-coding/intronic SNPs at the four AMD-associated novel loci identified using FUMA^80,88^. Using the eQTL analysis, we assessed whether gene expressions were affected by the lead SNPs. Genetic variants at four novel loci showed a similar LD pattern between East Asians and Europeans (Supplementary Fig. 13). For cis-SNP-gene pairs up to 1 Mb apart, we performed association tests across 53 human tissues in the GTEx dataset v.8^89^. A *P* value $=9.43\times {10}^{-4}$ was set to be statistically significant for the cis-eQTL association after multiple testing corrections ($0.05/53$).

### Functional studies in animal models

#### Animals

All experiments using animals were approved by the Institutional Animal Care and Use Committee of the Agency for Science, Technology, and Research (A*STAR) (IACUC, Protocol number: 181334) and SingHealth Experimental Medicine Centre (SEMC) (IACUC, Protocol number: 2018/SHS/1449) in Singapore. Animal care and procedures were performed in accordance with the Guide for Care and Use of Laboratory Animals from the US National Institutes of Health and the Statement for the Use of Animals in Ophthalmic and Vision Research from the Association for Research in Vision and Ophthalmology (ARVO). C57BL/6J mice were purchased from InVivos Pte Ltd., Singapore. All mice were housed in an environmentally controlled room ($22^\circ {{{{{\rm{C}}}}}}$, $40-60\%$ humidity, and a 12-h light cycle) with food and water available ad libitum. Eyes were enucleated at the experimental endpoints. The neuroretina or RPE/choroid tissue was dissected from the eye cup before being lysed for RNA extraction^33^.

#### Quantitative real-time PCR (qRT-PCR)

We tested selected genes and only included those expressed in mouse ocular tissues in subsequent diseased models. Total RNA was extracted from homogenized ocular tissues using NucleoSpin RNA, Mini kit (Macherey-Nagel, Düren, Germany) following manufacturers’ instructions. RNA concentrations were determined using a Nanodrop 2000C Spectrophotometer 19 (Thermo Fisher Scientific, Waltham, MA, USA). cDNA was synthesized using qScript cDNA Supermix (Quanta BioSciences, Beverly, MA, USA) according to the manufacturer’s instructions. Quantitative real-time PCR was performed in a total volume of $20\upmu {{{{{\rm{l}}}}}}$ containing PrecisionFAST 2× qPCR Mastermix (with SYBR green and low ROX) (Primerdesign, Camberley, UK) using LightCycler® 480 System (Roche, Basel, Switzerland). Data were analyzed using the ${2}^{-\Delta \Delta C(T)}$ method for quantification of the relative mRNA expression levels. The primer sequences used in this study were purchased from IDT and are listed in Supplementary Data 17. All gene expressions were normalized to that of GAPDH.

#### In silico analysis of transcription factor binding sites

The location of the top 30 SNPs sequences located at *GATA5* loci on chromosome 20 was analyzed using the PROMO v8.3 software (https://alggen.lsi.upc.es/cgi-bin/promo_v3/promo/promoinit.cgi?dirDB=TF_8.3), which incorporates TRANSFAC v6.4^90,91^, followed by analysis using FunciSNP (https://www.bioconductor.org/packages//2.12/bioc/html/FunciSNP.html)^92^. PROMO parameters were chosen for human TFBS. The SNP sequence carrying each allele was loaded as the query sequence. A schematic representation of the analysis output and the summary of TF where lead SNPs are located are shown in Supplementary Fig. 8.

#### Cells and cell culture

Primary human pigmented epithelial cells (HRPEs) were purchased from Angio-Proteomie (cAP-0037, Boston, MA, USA) and maintained in HRPECs Growth Medium (contains $10\%$ serum and growth supplements, cAP-33) following the manufacturer’s instructions. HRPE cells were plated in a 6-well plate and cultured for 24 h. Cells were washed once with PBS before being treated with MEM media (Lonza Bioscience) containing $10\upmu{{{{{\rm{M}}}}}}$ retinoic acid, dexamethasone, or vehicle control dimethyl sulfoxide (DMSO) for another 24 h^93^. Cells were washed three times with PBS before being lysed for total RNA extraction using NucleoSpin RNA, Mini Kit (Macherey-Nagel, Duren, Germany) following the manufacturer’s instructions. cDNA was synthesized using qscript cDNA Supermix (Quanta BioSciences, Beverly, MA, USA) according to the manufacturer’s instructions. Quantitative real-time PCR was performed in a total volume of $20\upmu {{{{{\rm{l}}}}}}$ containing PrecisionFast 2x qPCR Mastermix (with SYBR green and low ROX) (Primerdesign, Camberley, UK) using a lightCycler 480 (Roche).

#### Laser-induced CNV and RNA collection from RPE/choroid complex of the eye

Laser-induced CNV was performed as previously described^94^. In brief, a laser photocoagulator (HGM Medical Laser Systems Inc. Salt Lake City, USA) was used to induce CNV in mixed gender 6- to 8-week-old C57BL/6J mice. Six laser burns were generated in each eye ~1 disc diameter from the optic nerve. The laser settings used were $250{{{{{{\rm{mv}}}}}}}$ power, $0.1{{{{{\rm{s}}}}}}$ duration, and $100\upmu {{{{{\rm{m}}}}}}$ spot size. The eyes were enucleated on day 35 following the laser injury. RNA was extracted from the RRE/choroid tissues using the NAEasy mini kit (Qiagen, Hilden, Germany) and snap-frozen for gene expression analysis.

#### Statistical analyses

Data are represented as mean $\pm$ standard error (SEM). Statistical comparison of the results was performed via two-tailed, unpaired Student’s *t* test, one-way ANOVA, or Dunnett’s multiple comparisons test, using Prism 8.0 (GraphPad, La Jolla, CA, USA; https://www.graphpad.com/). Statistical significance is denoted with asterisks as follows: $*{P}\, < \,0.05$; $*\ast {P}\, < \,0.01$; $*\ast*P\, < \,0.001$ and $*\ast*\ast P\, < \,0.0001$.

### Reporting summary

Further information on research design is available in the Nature Portfolio Reporting Summary linked to this article.

### Supplementary information

Supplementary Information

Description of Additional Supplementary Files

Supplementary Data 1-17

Reporting Summary

### Source data

Source Data

## Data Availability

The summary statistics of the top index variants were presented in the Supplementary Data, along with the data analyzed in this study. The summary statistics file from the meta-analysis for nAMD from GAMA is available on the Amazon Web Services (AWS) from https://gamagentics.s3.ap-southeast-1.amazonaws.com/AMD_Topvariants_EAS_NC2023.txt. To protect patient privacy, individual-level whole-exome sequencing data access is restricted. Those interested in accessing the data are encouraged to contact corresponding authors to discuss potential collaborations and sign data use agreements. All requests for data access will be reviewed by our internal committee. Responses will be provided within 4 weeks. All datasets generated during the study can be obtained by contacting the corresponding authors. The IAMDGC AMD GWAS results are downloaded from the GWAS Catalog http://ftp.ebi.ac.uk/pub/databases/gwas/summary_statistics/GCST003001-GCST004000/GCST003219/ under study accession identifier GCST003219. The 1000 Genomes Phase 3 data on Genome Reference Consortium Human Build 37 (GRCh37) is available at https://www.internationalgenome.org/. The datasets used for eQTL mapping and gene-set analysis through the FUMA platform (https://fuma.ctglab.nl/) are available through: GTEx eQTL v8 (https://www.gtexportal.org/home/datasets) and MSigDB v7.0 gene-set file (https://www.gsea-msigdb.org/gsea/msigdb). Source data supporting our findings (Fig. 3 and Supplementary Figs. 8, 9 and 12) were provided in this paper as a Source Data file. Source data are provided with this paper.
