# Supplementary material for: Contribution of common and rare variants to Asian neovascular age-related macular degeneration subtypes
Source: Nat Commun. 2023 Sep 11;14:5574. doi: 10.1038/s41467-023-41256-z (PMC10495468; doi:10.1038/s41467-023-41256-z)
Supplement: Supplementary file 1 — Supplementary Information [file 41467_2023_41256_MOESM1_ESM.pdf]

Supplementary Information

Contribution of common and rare variants to Asian neovascular age-related macular degeneration subtypes

Qiao Fan et al

Supplementary Notes

Supplementary Note 1: GWAS/WES cohorts
Supplementary Note 2: Ethnics oversight

Supplementary Figures

Supplementary Figure 1: Flow chart of cases and controls included for each study for GWAS and WES.
Supplementary Figure 2: Genetical structure of cases and controls using Principal Component Analysis.
Supplementary Figure 3: Quantile-quantile (QQ) plots for Asian nAMD GWAS and subtypes**.**
Supplementary Figure 4: Regional plots of lead variants.
Supplementary Figure 5: Concordance between WES and directly genotyped array.
Supplementary Figure 6: Manhattan plot and QQ plots for PCV versus typical nAMD.
Supplementary Figure 7: Regional plot of the top GWAS locus for PCV versus typical nAMD.
Supplementary Figure 8: qRT-PCR analysis of the expression of various genes in RPE/Choroid complex of mouse eye at P4, P14 and P28 respectively.
Supplementary Figure 9: qRT-PCR analysis of *GATA5* expression in ocular tissues and Human RPE.
Supplementary Figure 10: In silico analysis of transcription binding sites of top 30 SNPs.
Supplementary Figure 11: Human RPE morphology before and after retinoic acid treatment
Supplementary Figure 12: mRNA expression in human HRPE cells upon dexamethasone treatment.
Supplementary Figure 13: Coverage of target samples in whole-exome sequencing.
Supplementary Figure 14: Pair-wise LD heatmap at four novel loci in East Asians and Europeans.

Supplementary Notes

Supplementary Note 1: GWAS/WES cohorts

Singapore

Cases: The age-related macular degeneration (AMD) cases were recruited from the retinal clinics of three major public hospitals with tertiary eye care in Singapore, including the Singapore National Eye Center, National University Health System, and Tan Tock Seng Hospital, between September 2007 and April 2019[1]. AMD status was accessed based on retinal photos following the Wisconsin Age-Related Maculopathy Grading System[2]. Patients underwent a comprehensive ocular examination, including dilated fundus examination, fundus angiography with fluorescein and indocyanine green (ICG) using a fundus camera (TRC50X/IMAGEnet 2000, Topcon, Tokyo, Japan) or confocal scanning laser ophthalmoscope (Spectralis, Heidelberg Engineering, Heidelberg, Germany), and optical coherence tomography (OCT) (Cirrus, Carl Zeiss Meditec, Dublin, California, USA). For each sample, $10 - 20mL$ of venous blood was collected for DNA extraction for genotyping purposes. We only included patients of Chinese ethnicity in this study.

Controls: Controls were Chinese participants aged 40 years and older ascertained from the Singapore Chinese Eye Study (SCES)[3]. The SCES is a population-based cohort study of Chinese adults residing in the southwestern part of Singapore. The Ministry of Home Affairs of Singapore provided an initial computer-generated list of Chinese adults aged 40 to 80+ years old. A final sampling frame of 6,350 ethnic Chinese residents was derived from this list using an age-stratified random sampling strategy. A control was defined without any clinical signs of early or advanced AMD.

For GWAS, a total of 3,838 DNA samples (1,344 cases and 2,493 controls) were genotyped using Illumina OmniExpress or Human610-Quad BeadChip v1 on up to 714,255 variants.

For exome sequencing, 301 PCV cases were sequenced using NimbleGen SeqCap EZ (Roche) kit to enrich genomic DNA for coding regions, on the Illumina HiSeq 1000 platform (target $50\times$) using paired-end sequencing of $150\ bp$ or $100\ bp$. WES data for healthy SCES controls were obtained under the SingHealth Duke-NUS Institute of Precision Medicine (PRISM) program. For 797 healthy controls, WES was performed using the NimbleGen SeqCap EZ (Roche) kit to enrich genomic DNA for coding regions, followed by sequencing on the Illumina NovaSeq 6000 platform using $150\ bp$ paired-end sequencing. 37 controls with early AMD were excluded. After quality controls, 259 PCV cases and 760 controls were included for further analysis.

This research is supported by the Singapore Ministry of Health's National Medical Research Council under NMRC/OFLCG/004/2018. We are grateful to the study's participants and staff for the data collection. The computational work for this study was performed on resources of the National Supercomputing Centre, Singapore (https://www.nscc.sg).

Hong Kong

Cases: All participants were Han Chinese of Guangdong descent recruited at the Hong Kong Eye Hospital and the Prince of Wales Hospital Eye Center, Hong Kong. Subjects with AMD in at least one eye and without other causes of choroidal neovascularization (e.g., myopic maculopathy) in any eye were classified as cases in our study[4]. The clinical diagnosis and categorization of AMD followed the Age-Related Eye Disease Study (AREDS) criteria[5], based on results from ophthalmic examinations, including best-corrected visual acuity measurement, ocular tonometry, slit-lamp biomicroscopy, and color fundus photographs, as well as fluorescein angiography and high-speed confocal ICG angiography tests (Specrtalis, Heidelberg Engineering, Heidelberg, Germany).

Controls: The controls were Chinese participants from Hong Kong and Guangdong province[4]. The Hong Kong control participants aged 60 years or older were recruited from the Prince of Wales Hospital and the Hong Kong Eye Hospital. Those with major eye diseases (e.g., AMD) were excluded. Other healthy control participants aged a mean of 47 represented population-based controls (AMD not examined) in local communities in Guangdong province, China, as previously described[6,7].

For all study subjects, venous blood was collected to extract DNA. For GWAS, DNA samples were genotyped using Illumina OmniExpress-12 v1 for 520 cases on 714,255 variants and Human610-Quad BeadChip for 1,044 controls on 573,871 variants. We acknowledge the funding from the Direct Grants of the Chinese University of Hong Kong (4054119, CPP; and 2015.1.045, LJC; Hong Kong).

Japan

Cases: All AMD cases were of Japanese descent recruited from the Department of Ophthalmology at Kyoto University Hospital, Fukushima Medical University Hospital, and the Kobe City Medical Center General Hospital. All participants underwent a comprehensive ophthalmic examination, including determination of best-corrected visual acuity, intraocular pressure measurement, indirect ophthalmoscopy, slit-lamp biomicroscopy with a contact lens, and OCT examination (Spectralis, Heidelberg Engineering, Heidelberg, Germany; and/or Cirrus, Carl Zeiss Meditec, Dublin, California, USA). Subsequently, fluorescein angiography and ICG angiography were performed on each patient on fundus photographs, using a confocal laser scanning system (Heidelberg Retina Angiography II, Heidelberg Engineering, Heidelberg, Germany). Venous blood was collected to extract DNA for genotyping.

Controls: The control subjects were cataract patients without signs of AMD in both eyes and were recruited from the Department of Ophthalmology at Kyoto University Hospital, the Ozaki Eye Hospital, Mizoguchi Eye Clinic, the Japanese Red Cross Otsu Hospital, and Nagahama City Hospital.

DNA samples of 997 cases and 1,174 controls were genotyped using Illumina OmniExpress-12 v1 on 714,255 variants. We acknowledge grants-in-aid for scientific research (No. 24249082) from the Japan Society for the Promotion of Science, Tokyo, Japan.

Korea

Cases: Unrelated subjects of native Korean descent, aged 50 years or older, were recruited from 6 University Hospital-based tertiary retinal care centres, including the Seoul National University Bundang Hospital in Seongnam, Seoul National University Hospital in Seoul, Kyungpook National University Hospital, and Yeungnam University Hospital in Daegu, as well as Kosin University Hospital and Busan Paik Hospital in Busan, Korea. Each AMD patient was examined based on a standardized protocol to capture clinical, imaging and functional data. Based on clinical and ocular examination results, patients were categorized according to the AREDS classification system[5]. Late AMD was diagnosed and evaluated with fundus photographs, fluorescein and ICG angiography, and was categorized into typical neovascular AMD, polypoidal choroidal vasculopathy and geographic atrophy according to examination findings. Subjects with geographic atrophy were excluded from the current study. A $10 - 20\ ml$ sample of venous blood was collected to extract DNA for genotyping. The standard ocular examinations included dilated fundus examination, fundus photography, OCT (Spectralis, Heidelberg Engineering, Heidelberg, Germany; and/or Cirrus, Carl Zeiss Meditec, Dublin, California, USA) fundus fluorescein angiography and ICG angiography (Heidelberg Retina Angiography, Heidelberg Engineering, Heidelberg, Germany).

Controls: The hospital-based controls were from Seoul National University Bundang Hospital and other institutions in Korea. The average age was around 70 years (SD $= 9$). Subjects without any sign of AMD were enrolled as controls. They had no drusen and pigment abnormalities in the fundus photograph and/or optical coherence tomography.

DNA samples of 529 cases and 940 controls were genotyped using Illumina OmniExpress-12 v1 on 714,255 variants. We acknowledge the Seoul National University Bundang Hospital Research Grant Fund (grant no. 03-2009-008), and National Research Foundation of Korea (NRF-2009- 0072603, NRF-2012R1A1A2008943, NRF-2014R1A2A1A09005824) grants funded by the Ministry of Education, Science and Technology, Korea.

Supplementary Note 2: Ethnics oversight

All studies were performed with the approval of their Human Research and Ethics Committee, adhering to the Declaration of Helsinki principles. Written informed consent was obtained by the ethics committee of all the participating institutions as follows:

Singapore: Singapore National Eye Center, National University Health System, and Tan Tock Seng Hospital.

Hong Kong: Hong Kong Eye Hospital, Prince of Wales Hospital Eye Center, and Sun Yat-sen University Cancer Center.

Japan: Department of Ophthalmology at Kyoto University Hospital, Fukushima Medical University Hospital, Kobe City Medical Center General Hospital, Ozaki Eye Hospital, Mizoguchi Eye Clinic, Japanese Red Cross Otsu Hospital, and Nagahama City Hospital.

Korea: Seoul National University Bundang Hospital, Seoul National University Hospital, Kyungpook National University Hospital, Yeungnam University Hospital, Kosin University Hospital, and Busan Paik Hospital.

Supplementary Figures

Supplementary Figure 1: Flow chart of cases and controls included for each study for GWAS and WES.

Panel a) and b) depicts the workflow and sample size at every stage for GWAS and WES, respectively. nAMD: Neovascular AMD; PCV: polypoidal choroidal vasculopathy. Cases with other macular diseases such as central serous chorioretinopathy, high myopia, and angioid streaks were excluded. Controls were hospital-based controls without any clinical signs of AMD, or general controls enrolled from population-based studies. Quality Control (QC): For GWAS, samples with missing rate > 5% and heterozygosity > 6 standard deviations, related samples, ancestral outliers, and ambiguous phenotypes of cases were removed. For WES, we removed samples with missing rates > 10%, related samples, and ancestral outliers (see Methods).

a)

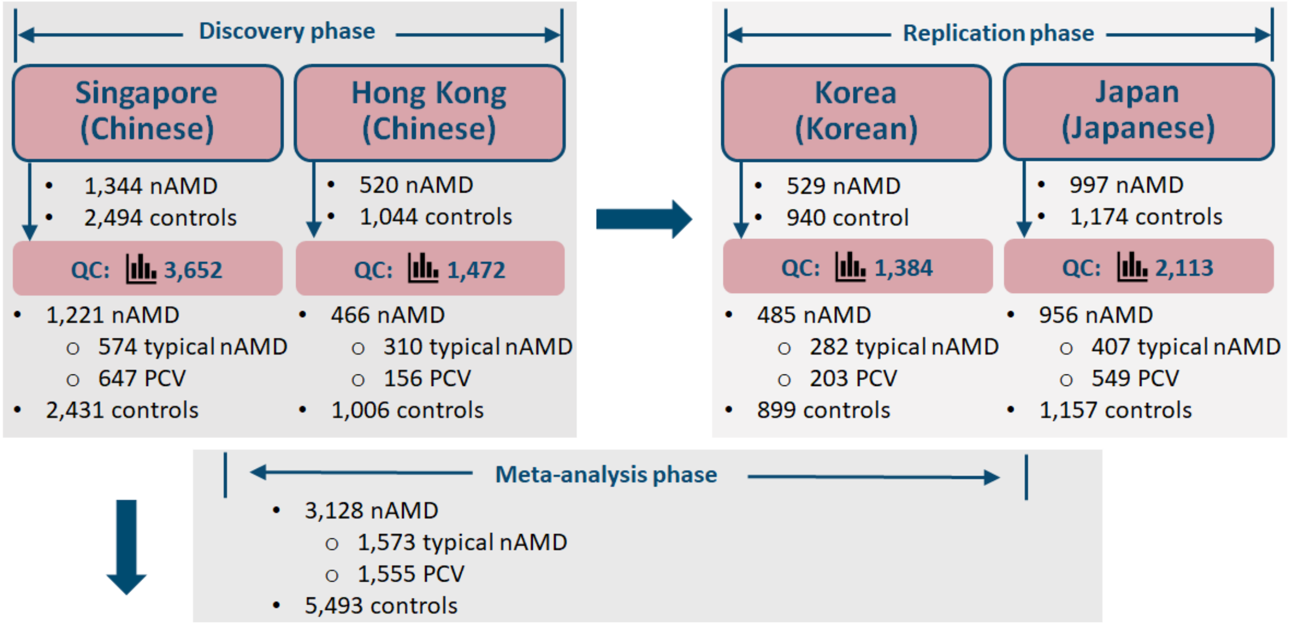

b)

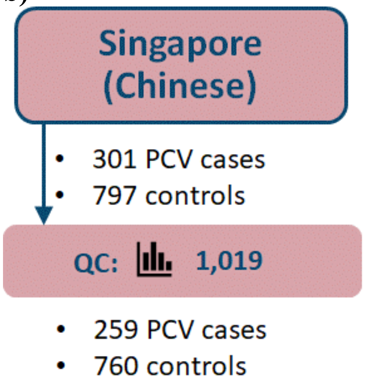

Supplementary Figure 2: Genetical structure of cases and controls using Principal Component Analysis.

Top-left and top-right figures are Hong Kong and Japan studies. Lower-left and lower-right figures are from Korea and Singapore (Chinese) studies. Red points for cases and blue points for controls. The first principal component (PC1) is on the x-axis and PC2 is on the y-axis. PCA was performed using directly genotyped data in each study, using EIGENSOFT version 7.2.1.

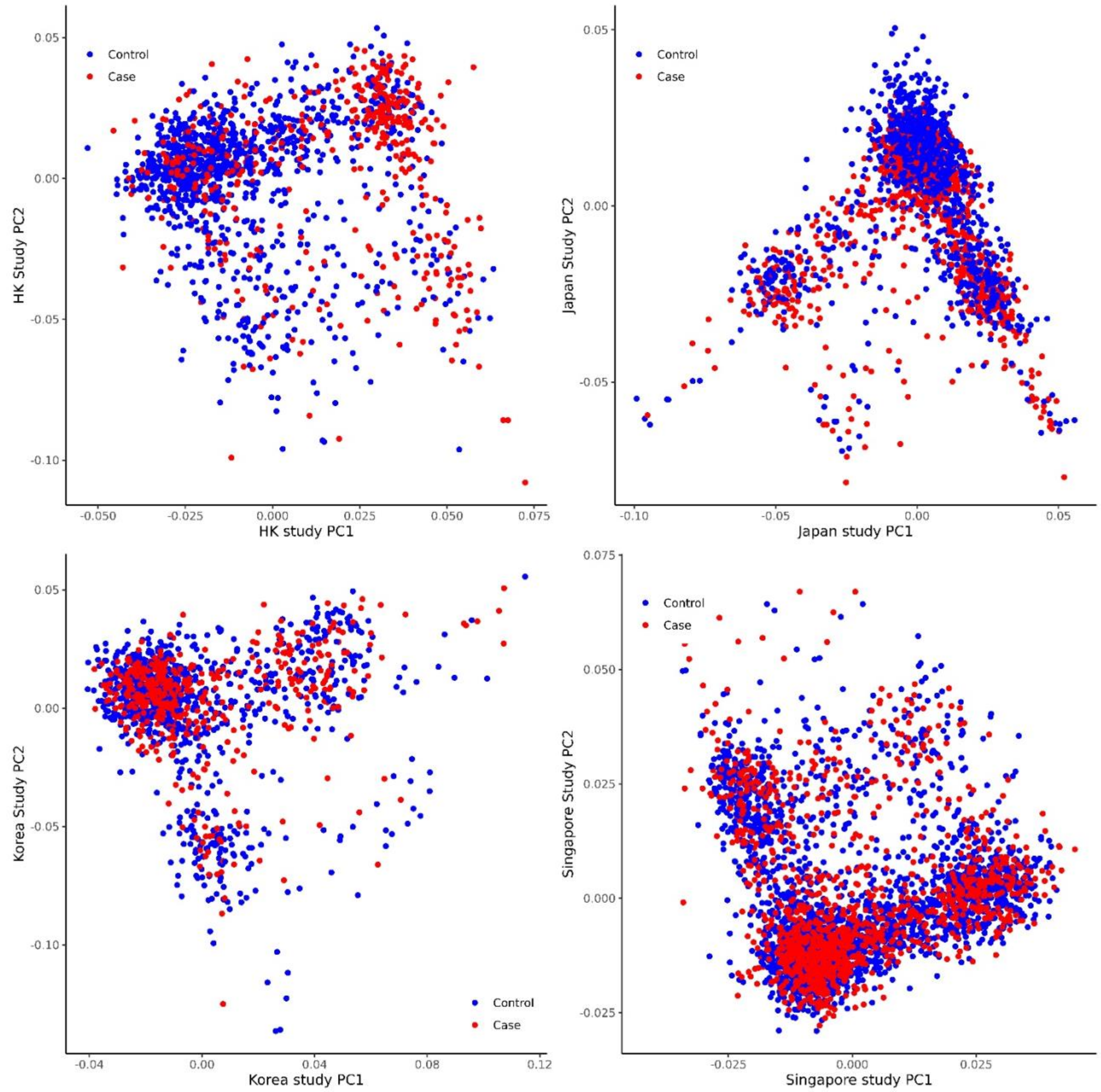

Supplementary Figure 3: Quantile-quantile (QQ) plots for Asian nAMD GWAS and subtypes.

GWAS discovery phase for nAMD contains 5,124 subjects. Meta-analyses of 4 cohorts for nAMD and two subtypes (PCV and typical nAMD) included a total of 8,621, 7,048, and 7,066 subjects, separately. We excluded variants with imputation quality indicator $r^2 < 0.5$ and minor allele frequency $< 0.1\%$ and kept only variants that exist in at least 3 out of 4 studies. Genomic inflation factor λ for the discovery phase and meta-analysis for nAMD, PCV, and typical nAMD GWAS are 0.99, 1.04, 1.06 and 1.02, separately. We determined λ by taking the median of χ^2 statistics for all variants and dividing it by the median of simulated χ^2 statistics at one degree-of-freedom.

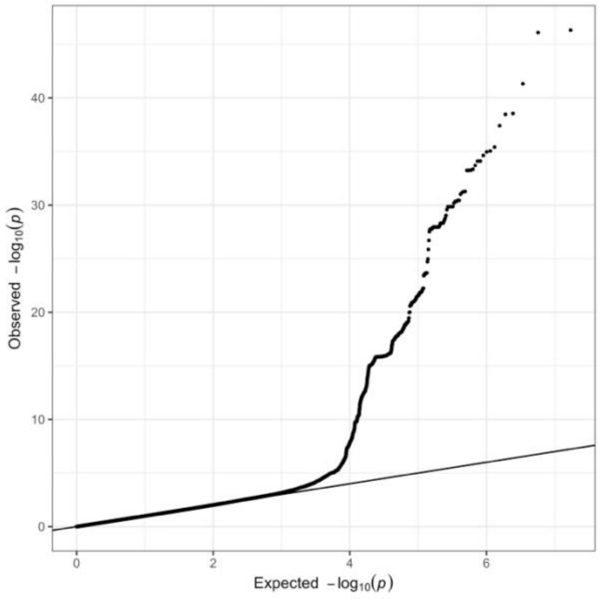

GWAS discovery phase for nAMD

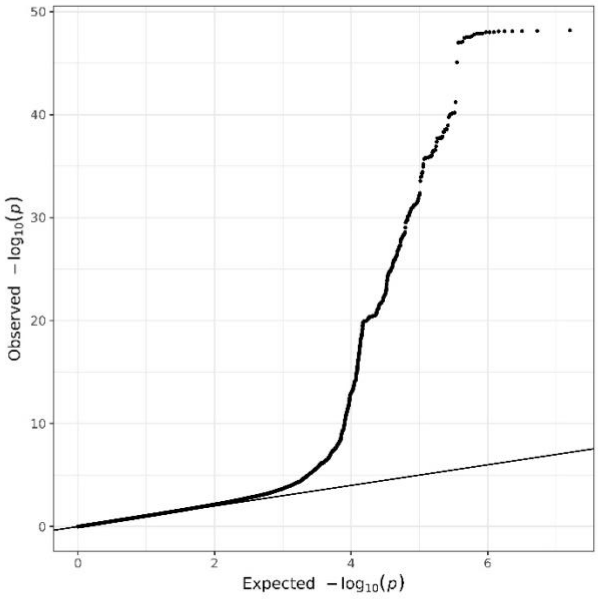

Meta-analysis for nAMD

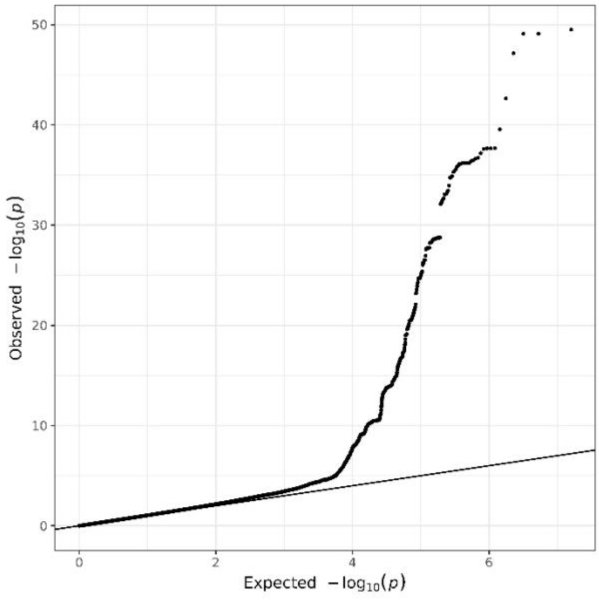

Meta-analysis for PCV

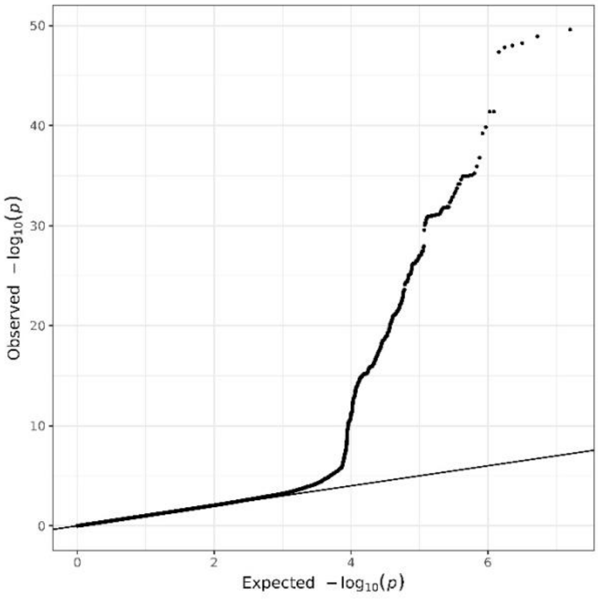

Meta-analysis for typical nAMD

Supplementary Figure 4: Regional plots of lead variants.

We made regional plots for 12 genome-wide significant lead SNPs from a meta-analysis using an online tool LocusZoom (http://locuszoom.org/). The default range covers 500 kb base pair of the lead variant's left or right flanks, i.e., a total of 1 Mb base positions were included. For each locus, we showed 3 regional plots for all nAMD and two subtypes (PCV and typical nAMD).

1. rs800292, chromosome 1, base pair 196642233, *CFH*

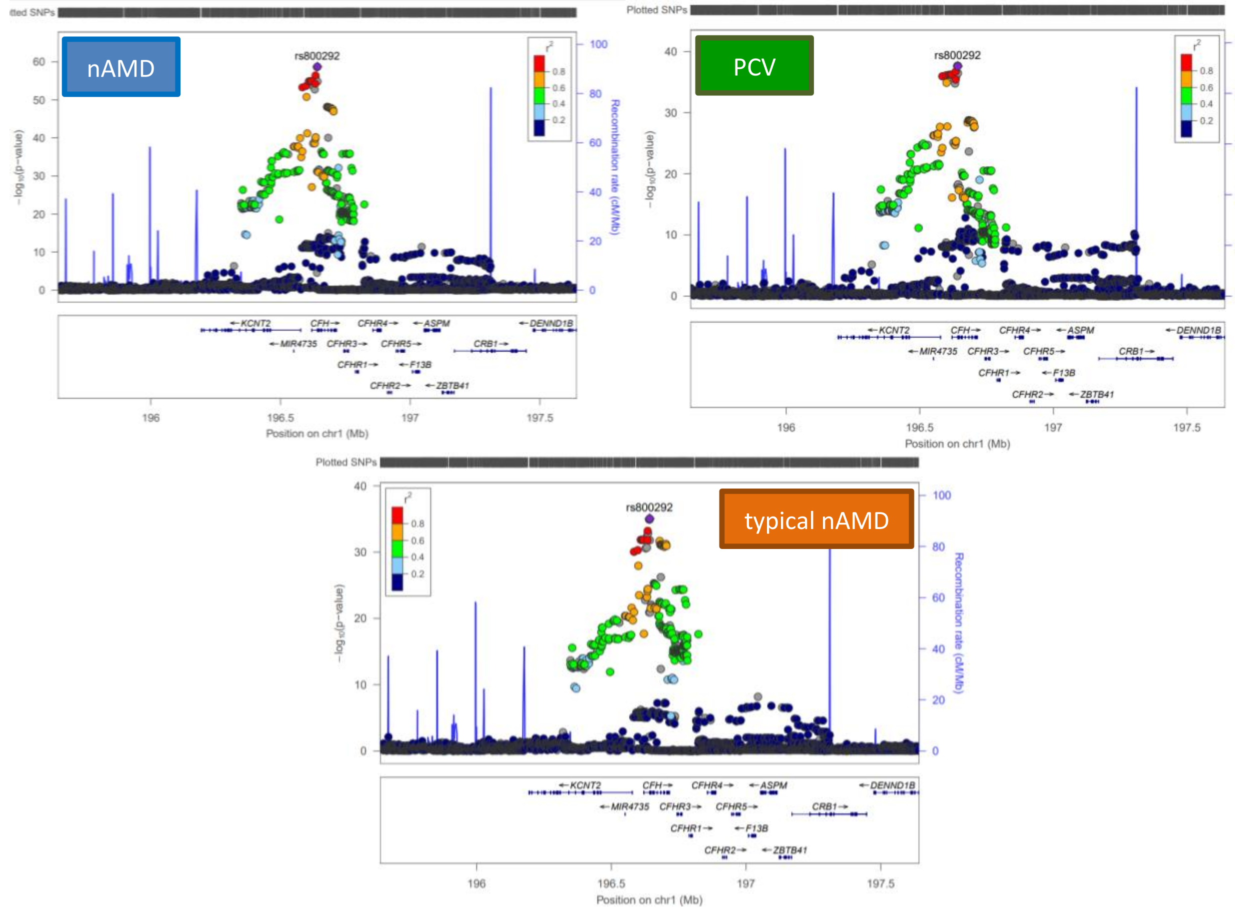

2. rs56033528, chromosome 2, base pair 227865660, *RHBDD1; COL4A4*

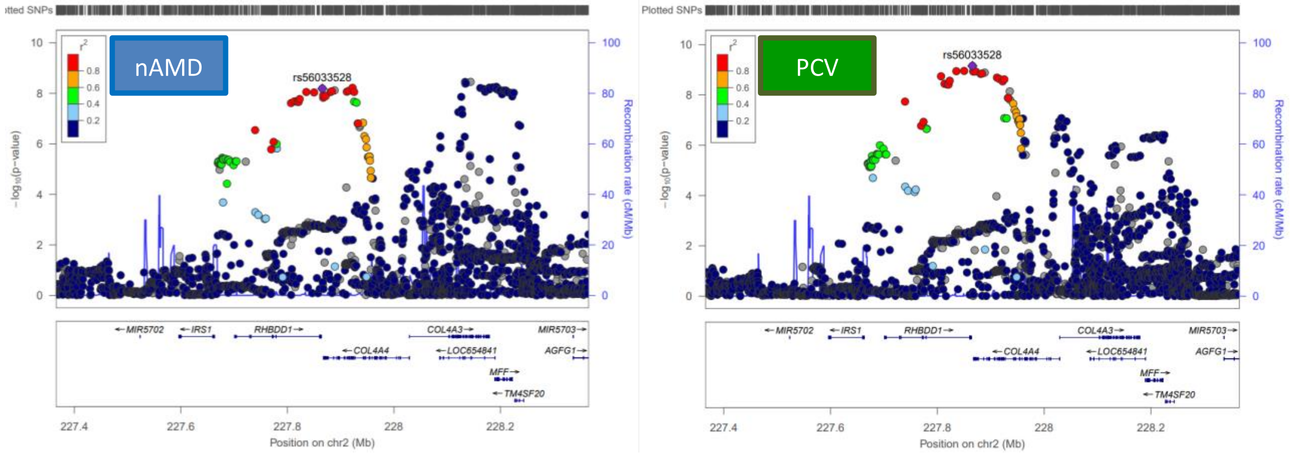

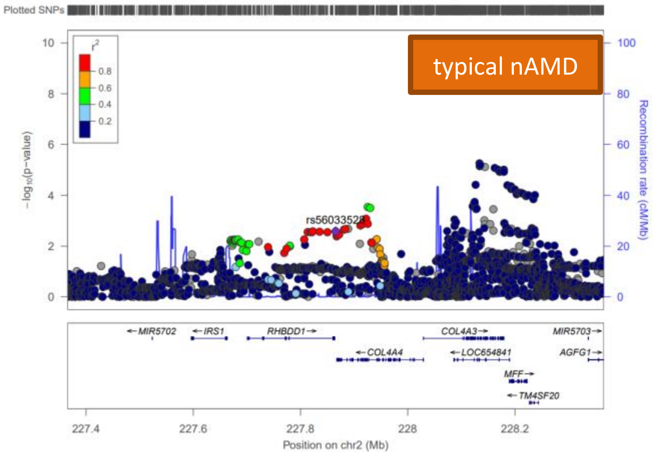

rs4643535, chromosome 2, base pair 228133809, *MFF; COL4A3*

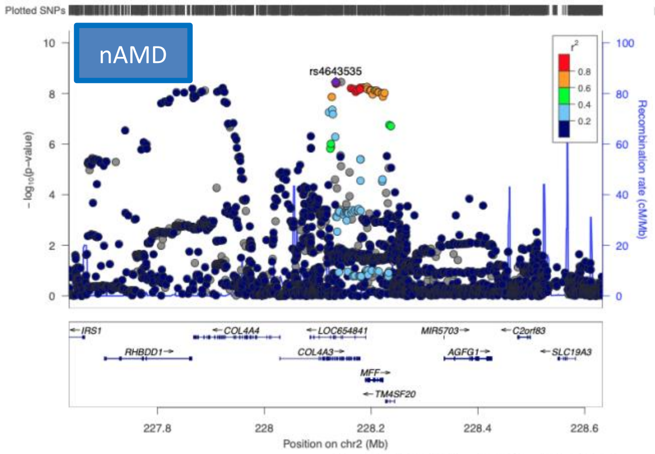

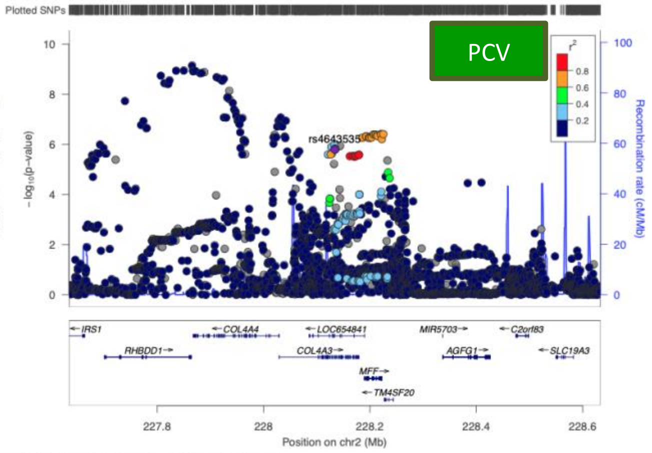

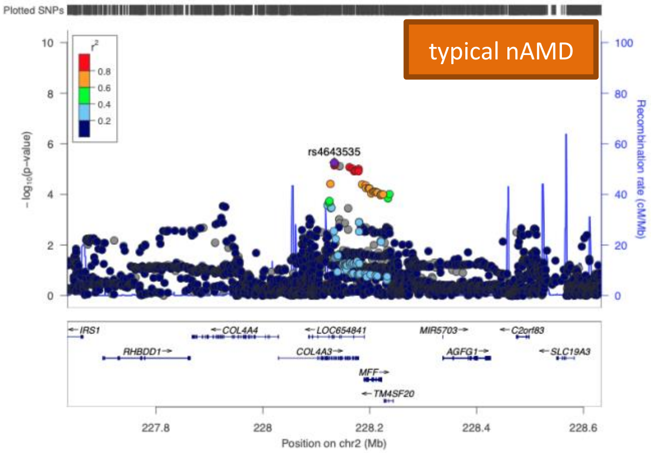

3. rs7428936, chromosome 3, base pair 64710850, *ADAMTS9-AS2*

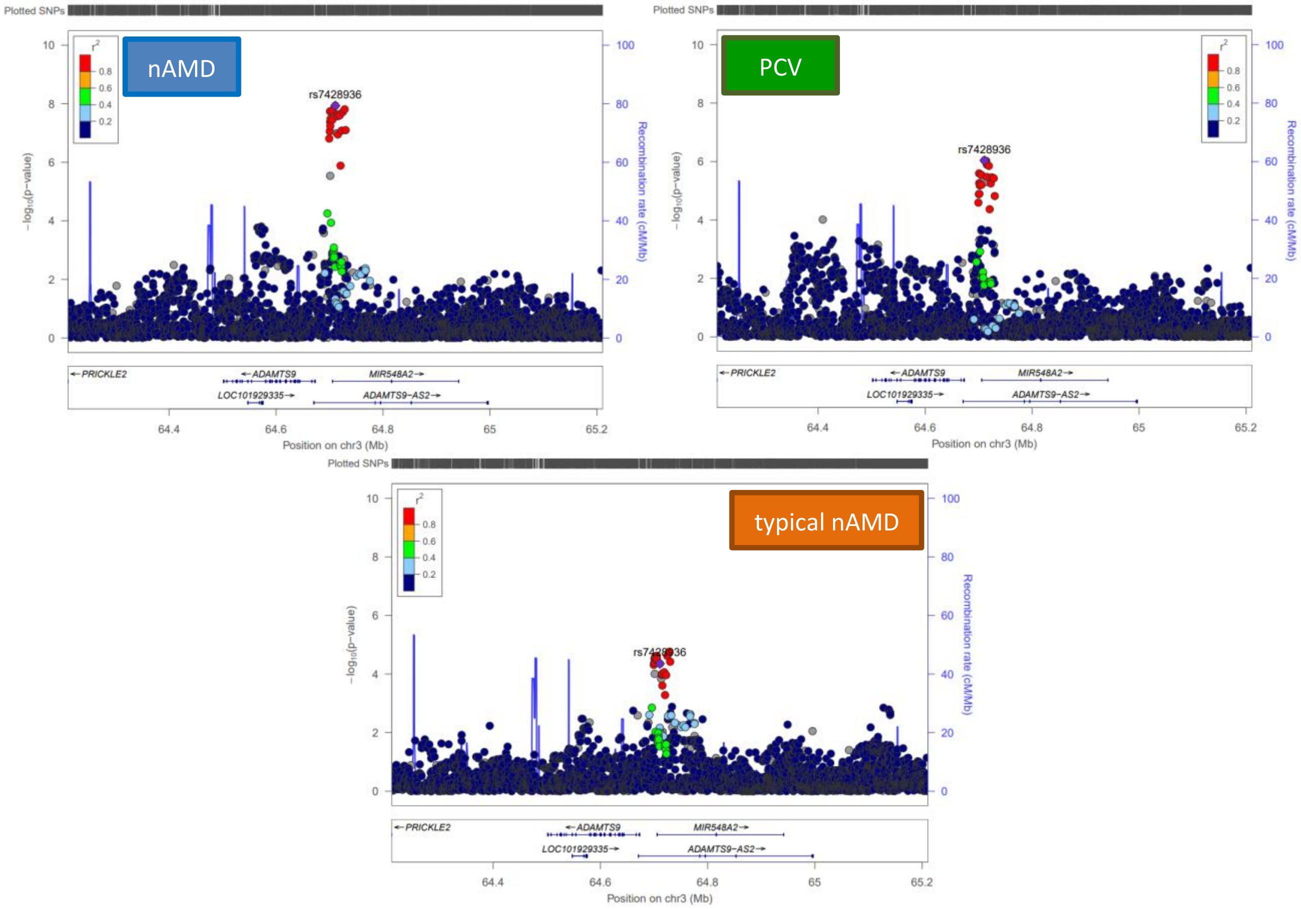

4. rs4151658, chromosome 6, base pair 31917557, *CFB*

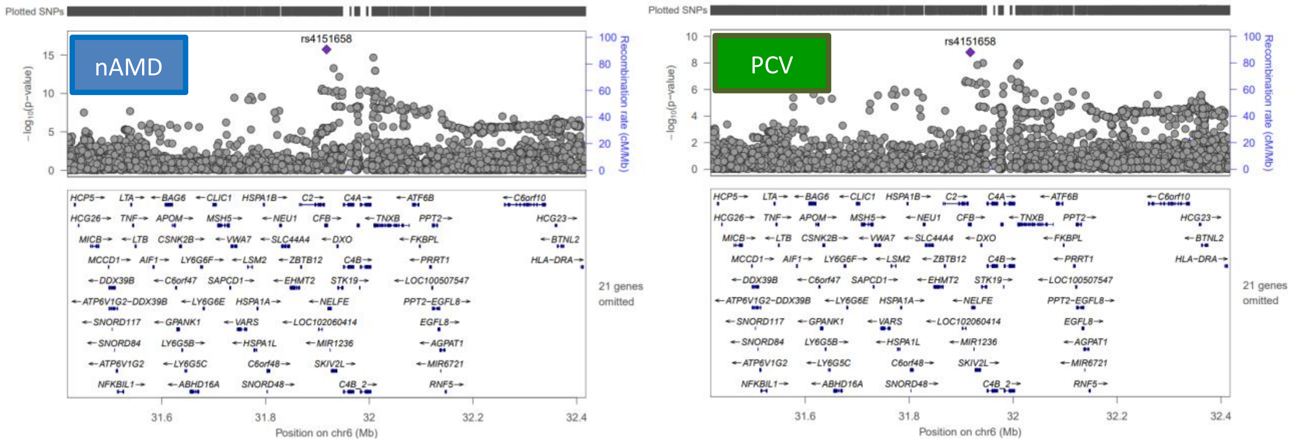

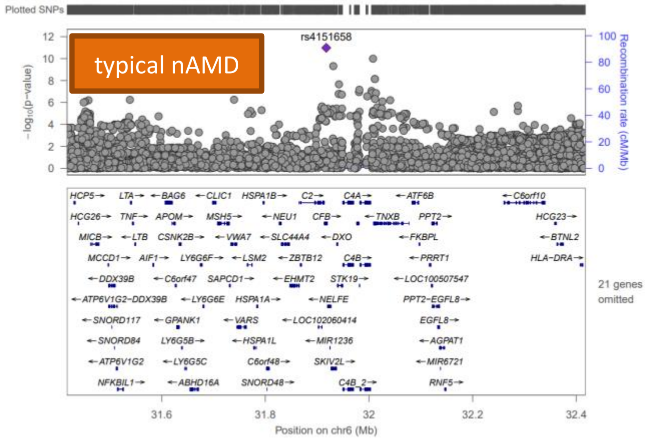

5. rs73733647, chromosome 6, base pair 43961015, *VEGFA; C6orf223*

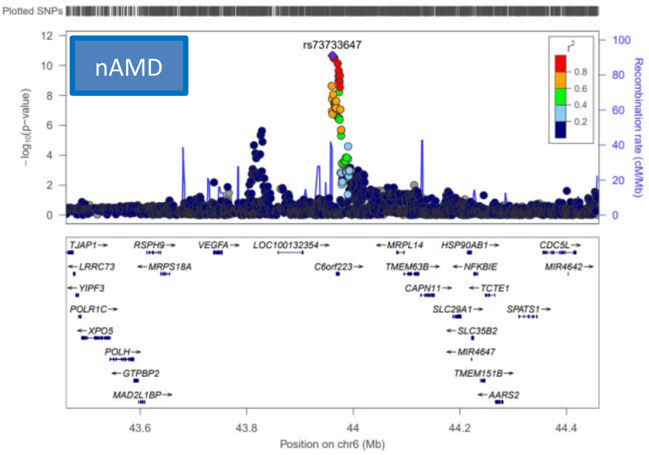

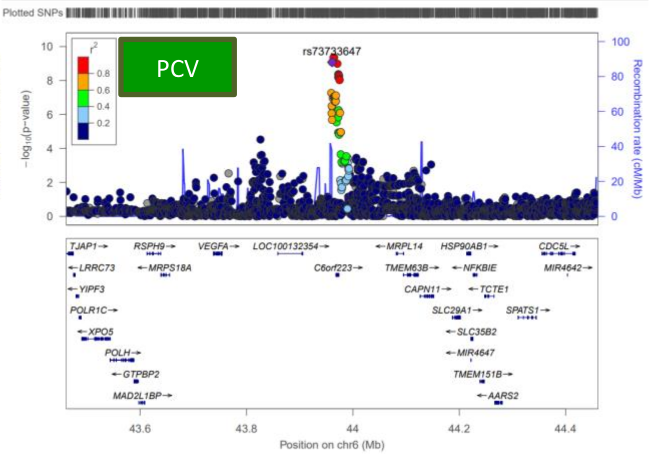

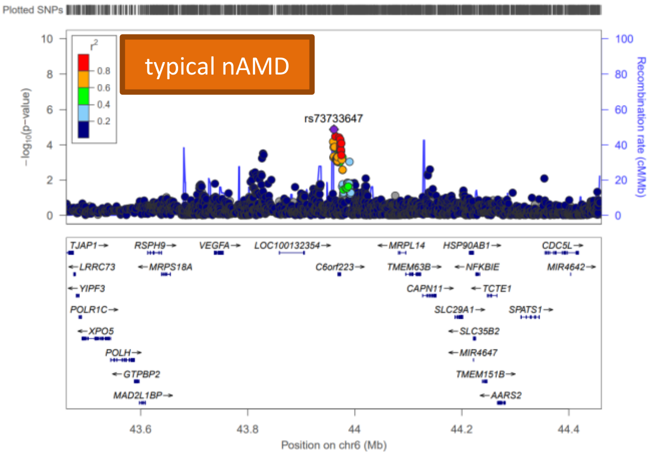

6. rs13278062, chromosome 8, base pair 23082971, *TNFRSF10A*
rs79037040 has merged into rs13278062

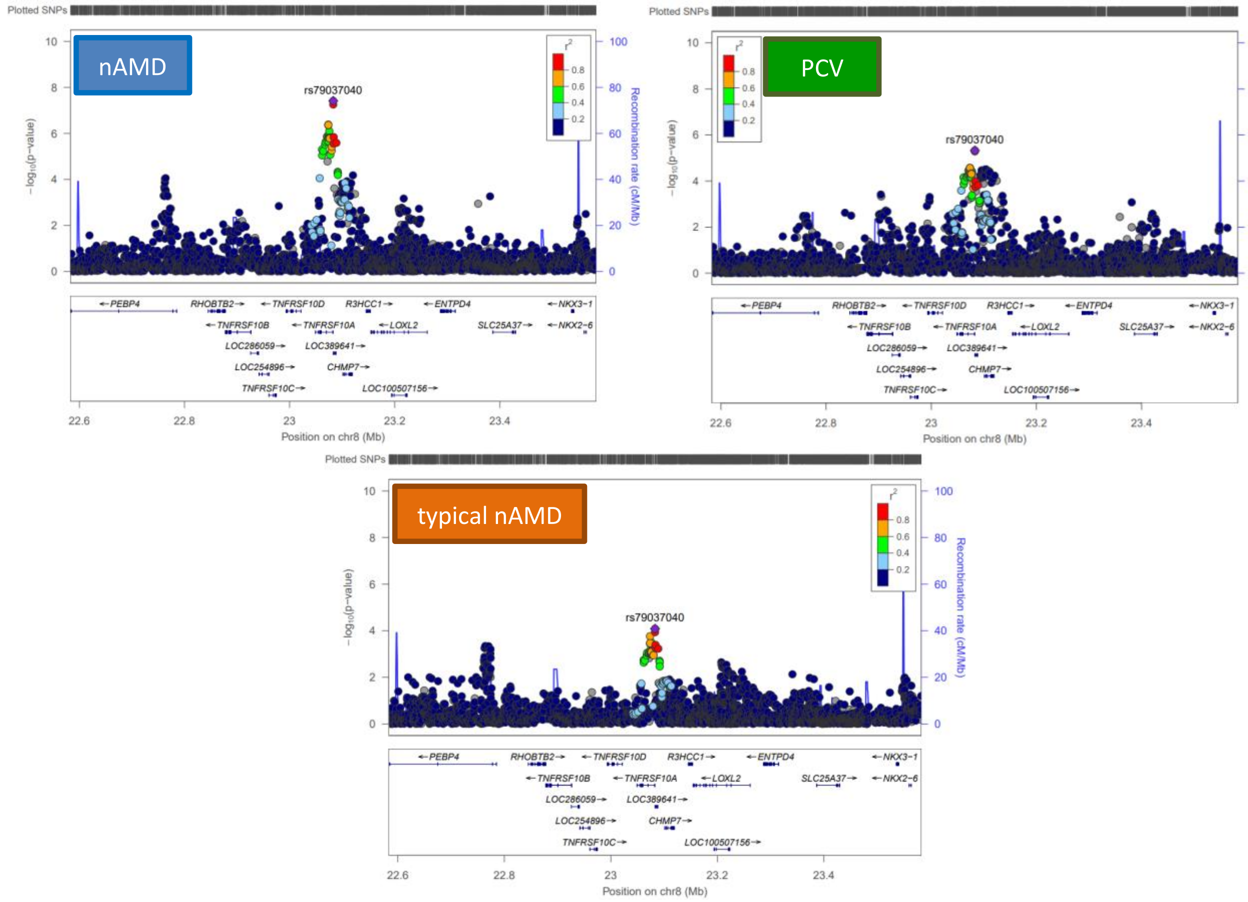

7. rs72759285, chromosome 9, base pair 126985763, *LHX2; NEK6*

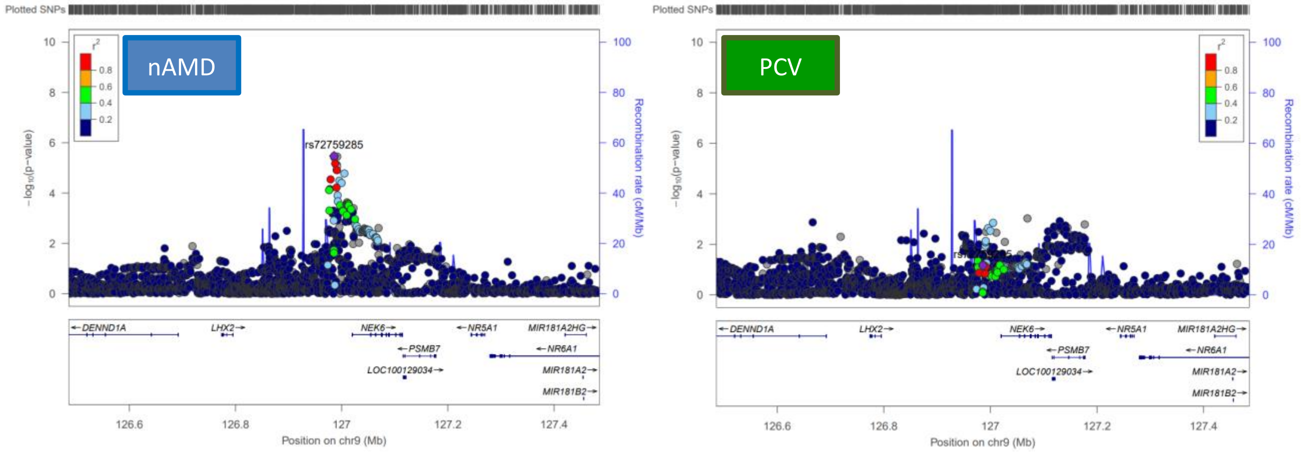

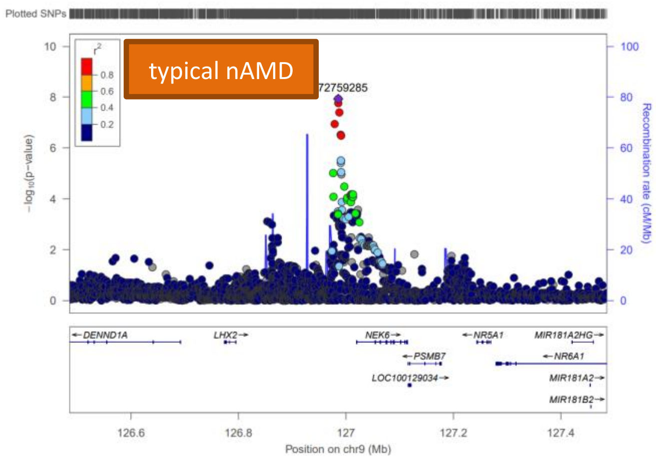

8. rs10490924, chromosome 10, base pair 124214448, *ARMS2*

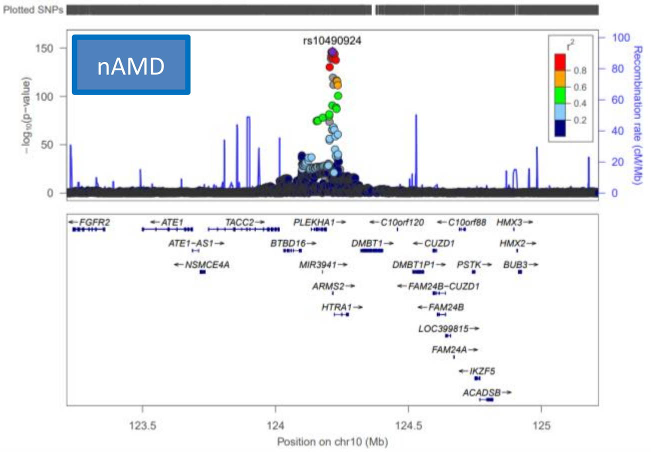

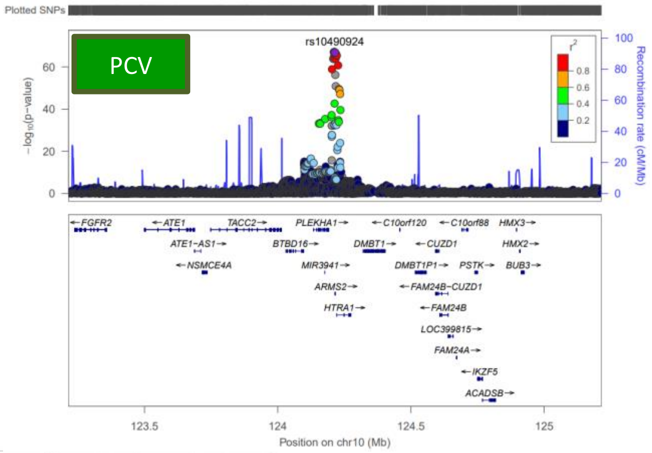

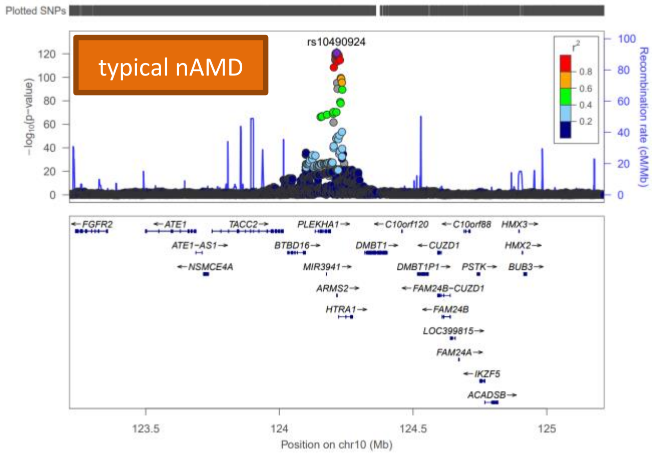

9. rs4769312, chromosome 13, base pair 24623220, *SPATA13*

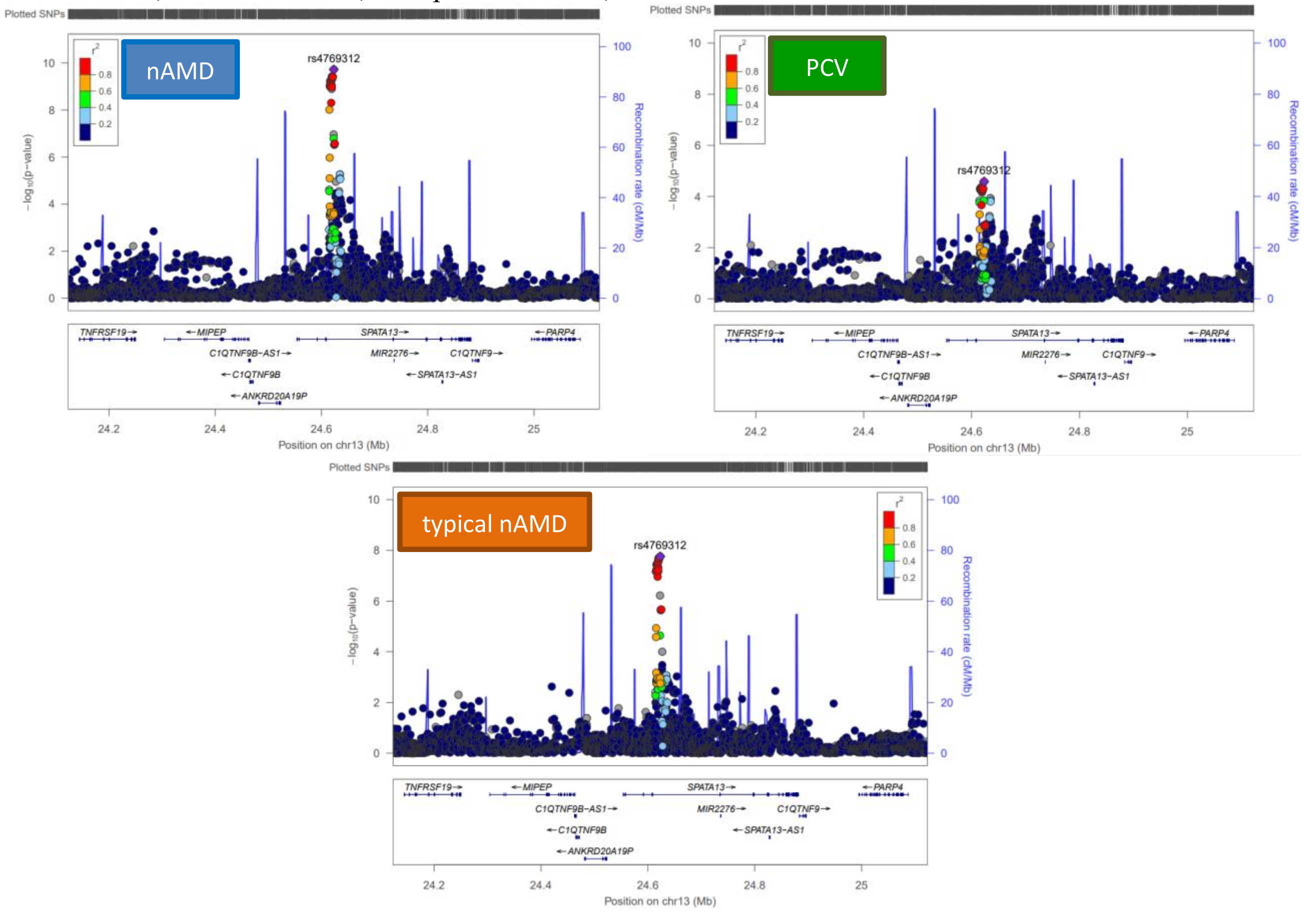

10. rs7402624, chromosome 15, base pair 102033538, *PCSK6; LINC02348*

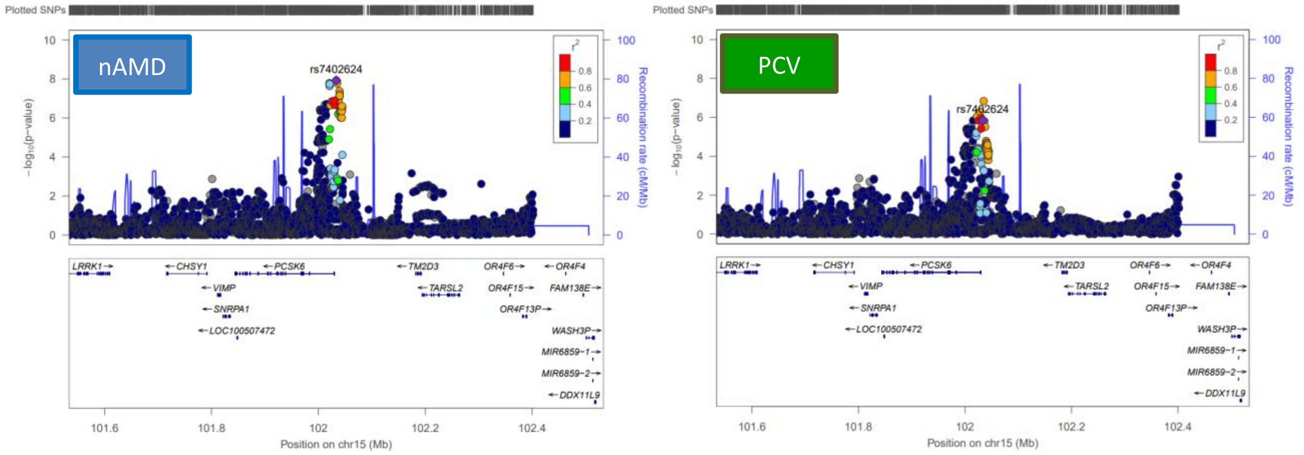

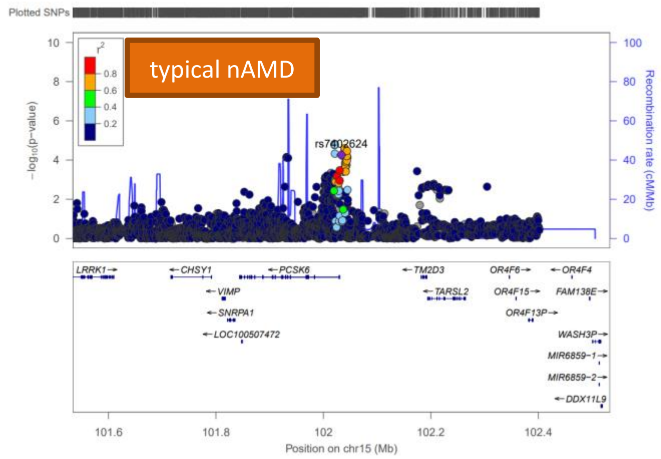

11. rs36229491, chromosome 16, base pair 56994244, *HERPUD1; CETP*

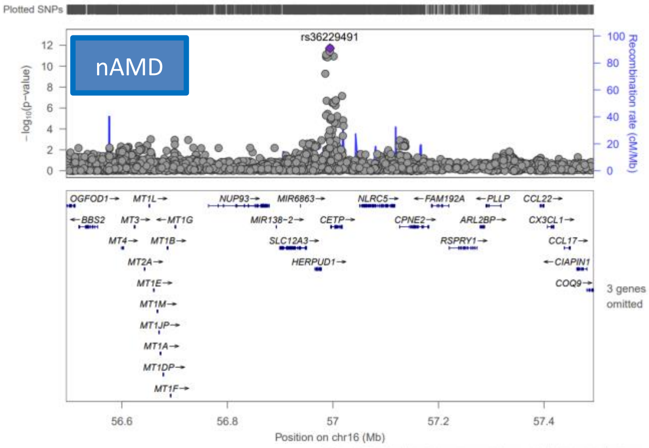

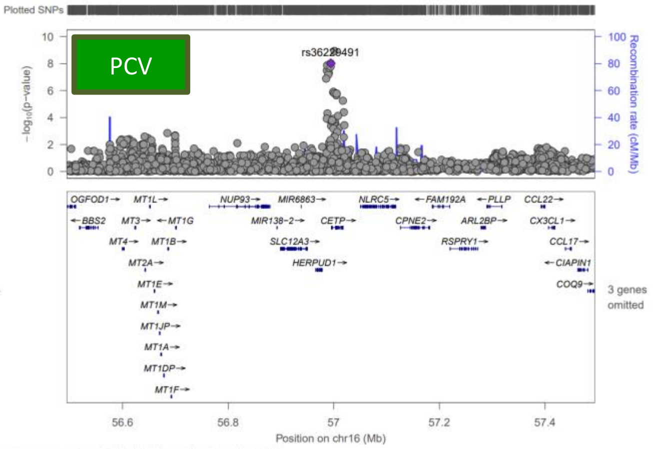

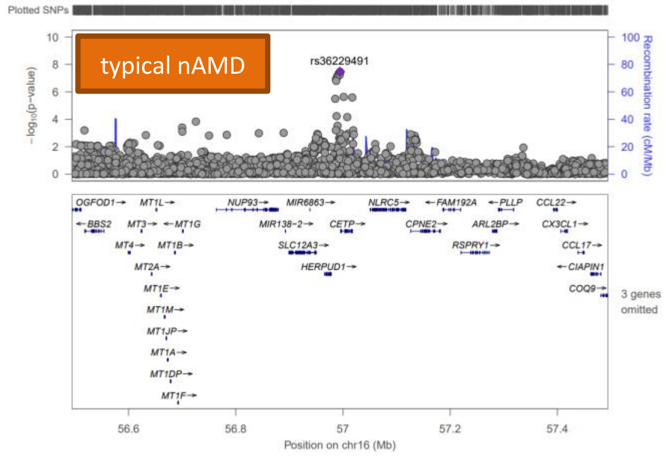

12. rs6121609, chromosome 20, base pair 61038322, *GATA5; LAMA5*

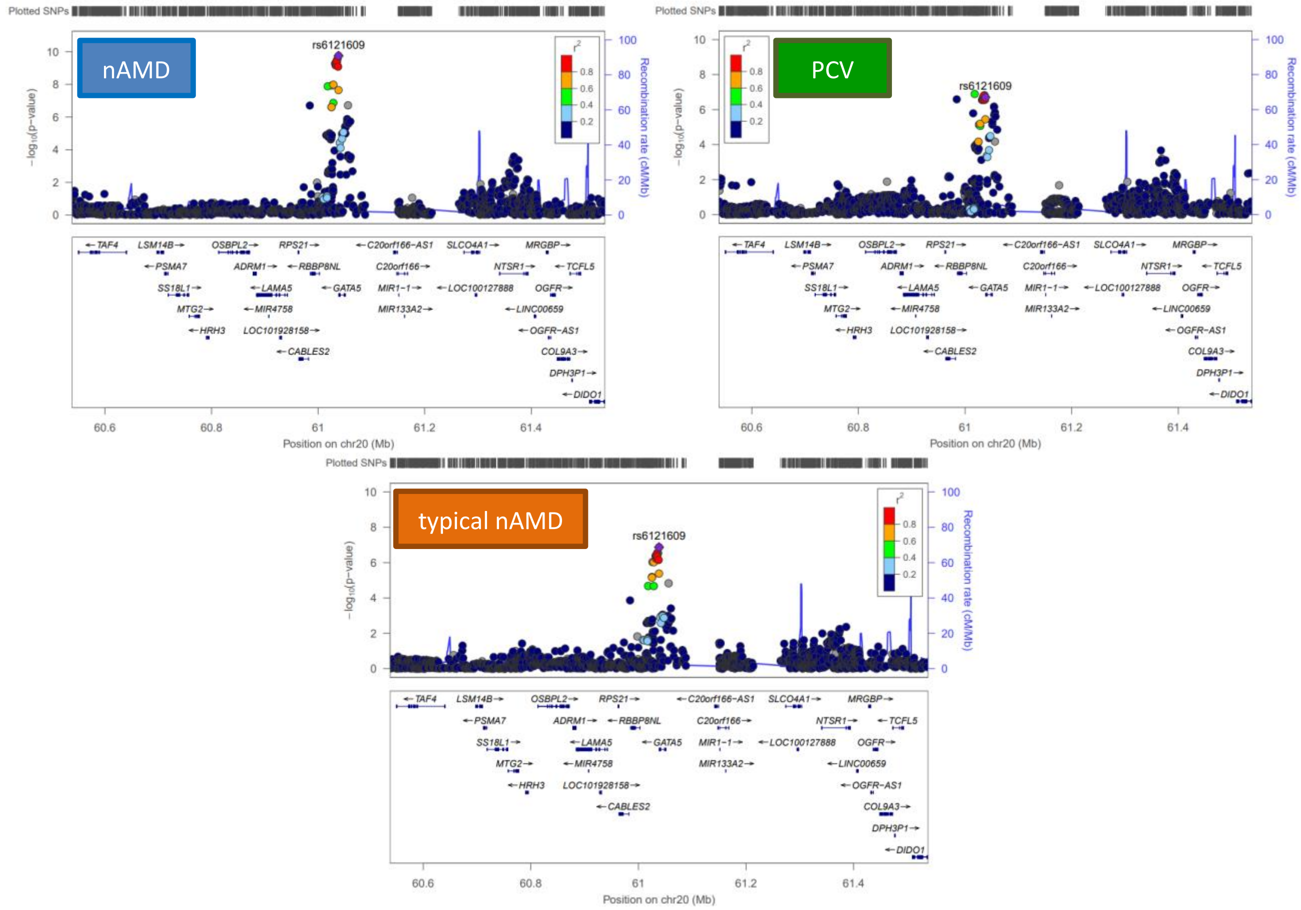

Supplementary Figure 5: Concordance between WES and directly genotyped array.

We measured concordance between WES and genotyped data in cases and controls, separately, using the squared correlation (R^2) of allele frequency grouped by three categories of MAF in WES data, i.e., MAF $\geq 5\%$, $1\% \leq$ MAF $< 5\%$ and MAF $< 1\%$. The concordance R^2 was computed based on the same set of samples, variants and alleles in WES and genotyped data for each category. This measure helps to interpret the assessment of accuracy for both rare and common variants. The concordance between WES and genotyped data were: $99.89\%/99.98\%$, $98.85\%/99.82\%$ and $95.09\%/98.61\%$ in cases or controls for variants of MAF in $5 - 50\%$, $1 - 5\%$ and $< 1\%$, respectively.

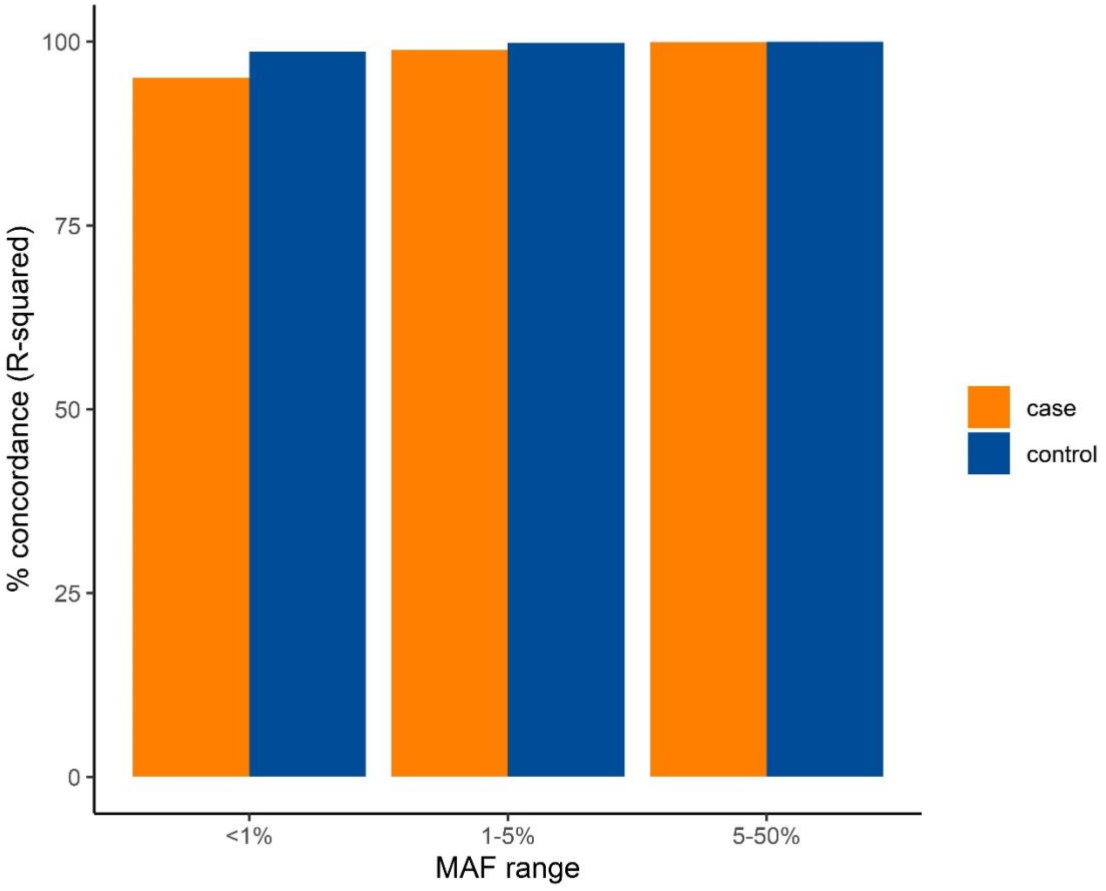

Supplementary Figure 6: Manhattan plot and QQ plots for PCV versus typical nAMD.

We carried out a GWAS analysis on each cohort and followed by a meta-analysis, using 1,555 PCV patients as cases and 1,573 typical nAMD patients as controls. A Firth bias-corrected likelihood-ratio model was used for a one-sided test using a χ^2 statistic with one degree-of-freedom. The meta-analysis was conducted under an inverse-weighted fixed-effect model. The red dashed line in the Manhattan plot (the upper figure) represents the genome-wide significance ($P = 5 \times 10^{-8}$). The genomic inflation factor lambda is 0.957.

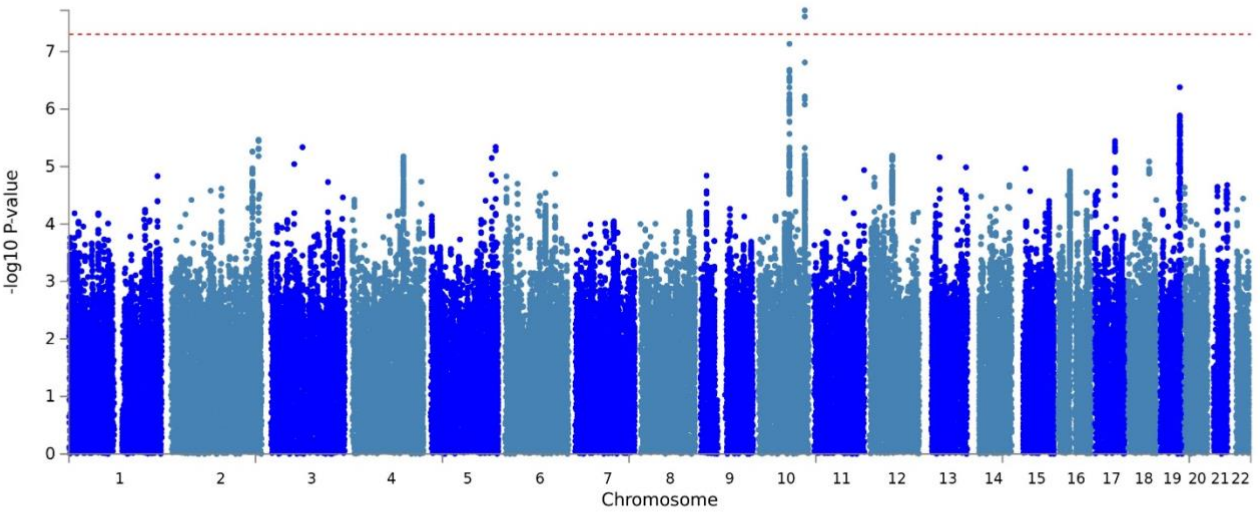

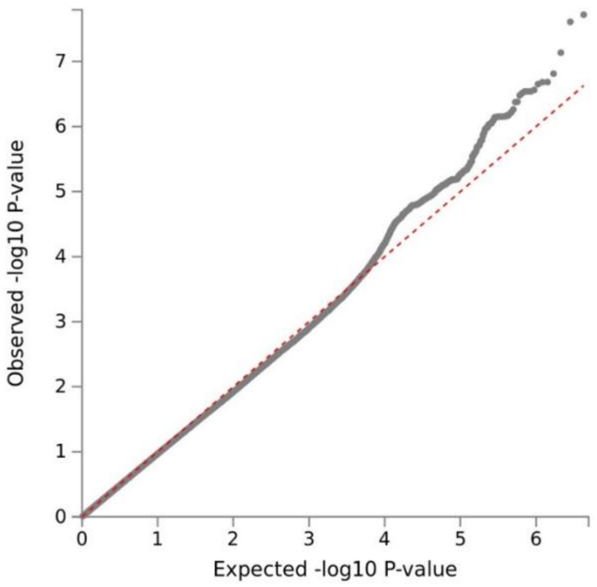

Supplementary Figure 7: Regional plot of the top GWAS locus for PCV versus typical nAMD.

Top variant rs61871744 (chr10:124203787) at *ARMS2* locus.

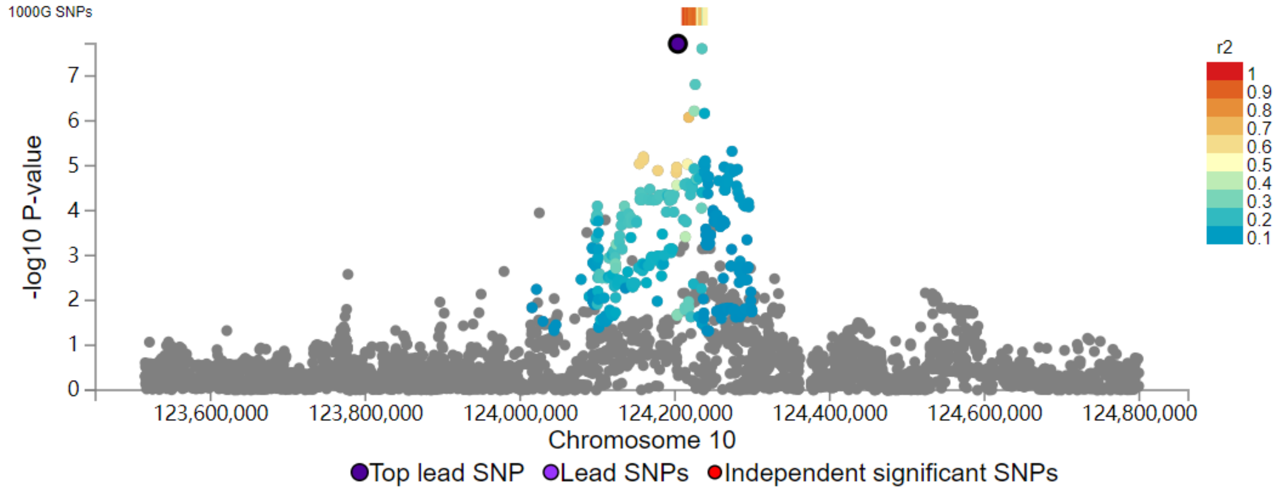

Supplementary Figure 8: qRT-PCR analysis of the expression of various genes in RPE/Choroid complex of mouse eye at P4, P14 and P28 respectively.

Data are expressed as mean $\pm$ S.D of $n = 3$ to 5 mice, specifically, *Vegfa* ($n = 3$ mice for all time points), *Col1a1* ($n = 5$ mice for P4 and P14, $n = 4$ mice for P28), *Mtg2* ($n = 4$ mice for P4 and P14, $n = 5$ mice for P28), *Lama5* ($n = 4$ mice for P4, $n = 5$ mice for P14 and P28), *Cables2* ($n = 4$ mice for P4, $n = 5$ mice for P14 and P28), *Col9a3* ($n = 4$ mice for P4, $n = 3$ mice for P14, and $n = 5$ mice for P28). One-way ANOVA was used to determine statistical significance.

For *Vegfa*, the *P*-value for P4 vs P14 is 0.0041; for P4 vs P21 is < 0.007. For *Col1a1*, the *P*-value for P4 vs P28 is 0.024 and P14 vs P28 is 0.005. For *Mtg2*, the difference between different treatment groups is not statistically significant. For *Lama5*, the *P*-value for P4 vs P14 is 0.021; for P4 vs P28 is 0.049. For *Cables2*, the *P*-value for P4 vs P14 and P4 vs P28 is < 0.0001. For *Col9a3*, the *P*-value for P4 vs P14 is 0.031; for P4 vs P28 is 0.048. Source data are provided in a Source Data file.

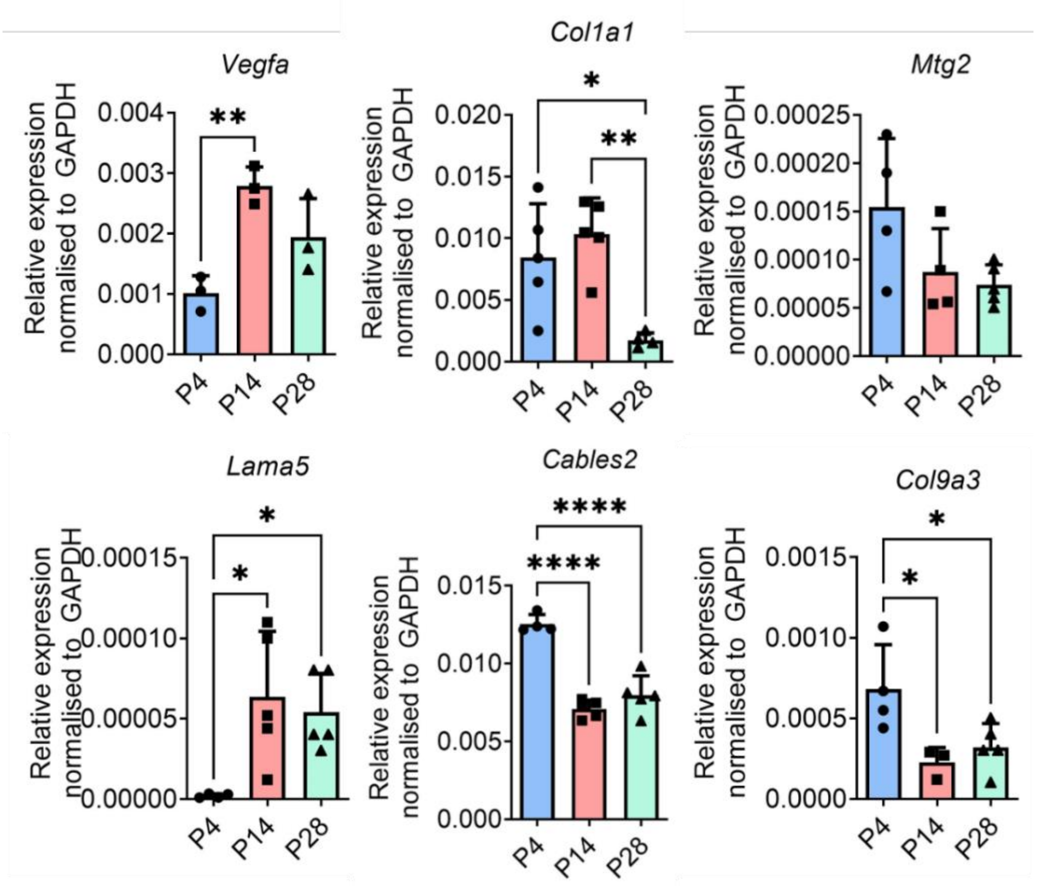

Supplementary Figure 9: qRT-PCR analysis of GATA5 expression in ocular tissues and Human RPE. a) in RPE/Choroid complex of mice at day 35 following the laser-induced choroidal neovascularization, and b) in human RPE cells subjected to RA treatment ($Ct > 30$).

Data are expressed as mean $\pm$ S.D of $n = 4$ mice. Source data are provided in a Source Data file.

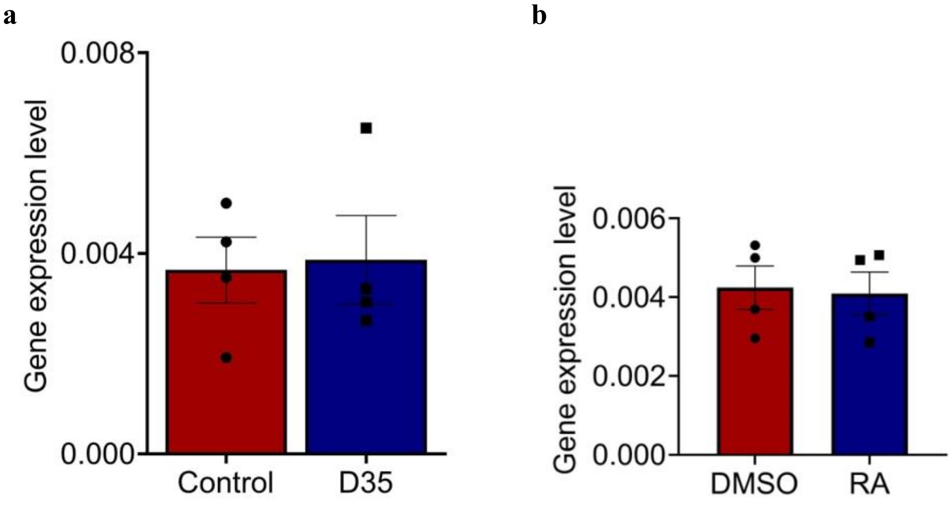

Supplementary Figure 10: In silico analysis of transcription binding sites of top 30 SNPs.

(a) Schematic representation of transcription factors binding prediction to sequences associated with SNPs genotypes located in the *GATA5* locus. Here we showcase SNP rs6089383 at chr20:61034707. SNP rs6089383 is the lead significant SNP with p-value at 3.70×10^{-10}, which is in strong LD with the lead index SNP rs6121609 ($r^2 = 0.942$). The transcription factor name with the database accession number in brackets; Dissimilarity (%), which corresponds to the rate of dissimilarity between the putative and consensus sequences for a given transcription factor; Random Expectation (RE) indicating the expected occurrences of the match in a random sequence of the same length as the query sequence according to the dissimilarity index, presented the RE equally (equiprobability for the four nucleotides) and RE query (nucleotide frequencies as in the query sequence). Markedly different changes are highlighted in grey and the SNP is highlighted in red. (b) Summary of transcription factors for all these SNPs examined with their key functions.

a.

rs6089383 (chr20:61034707)

CACTCGGCTGCCCT[G/A]CCCTCCCCACAGC

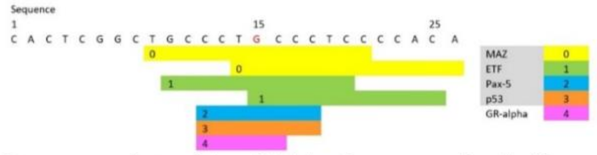

Factor name	Start position	End position	Dissimilarity	Sequence	RE equally	RE query
MAZ [T00490]	9	21	14.92	TGCCCTGCCCTCC	0.00083	0.07281
MAZ [T00490]	14	26	3.97	TGCCCTCCCCACA	0.00008	0.00535
ETF [T00270]	10	20	8.88	GCCCTGCCCTC	0.00031	0.00701
ETF [T00270]	15	25	5.25	GCCCTCCCCAC	0.00037	0.02575
Pax-5 [T00070]	12	18	0	CCTGCCC	0.01428	0.08246
p53 [T00671]	12	18	0	CCTGCCC	0.00476	0.02905
GR-alpha [T00337]	12	16	8.07	CCTGC	0.10156	0.14993

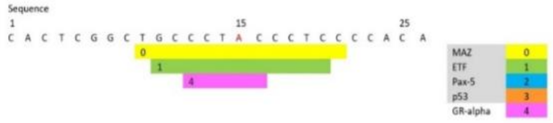

Factor name	Start position	End position	Dissimilarity	Sequence	RE equally	RE query
MAZ [T00490]	9	21	14.92	TGCCCTGCCCTCC	0.00083	0.07281
ETF [T00270]	10	20	8.88	GCCCTGCCCTC	0.00031	0.00701
GR-alpha [T00337]	12	16	8.07	CCTGC	0.10156	0.14993

b.

Transcription factor	Number of SNPs	Key functions
GR-Alpha	12	Inflammation and aging in the brain, macrophages, liver etc
RA/RXR	5	Retinoic acid-responsive transcription factors
PAX5	3	induced by RAR
E2F1	3	novel regulator of cell metabolism and regulates Retinoic acid mediated transcriptional activation
AP2alpha	3	A retinoic acid-responsive transcription factor, predominantly expressed in the retina, repress retinal FABP
EBF	3	regulate neuronal differentiation and genes involved in retinoic acid signalling
HNF-3alpha	2	A retinoic acid-responsive transcription factor
ENKTF-1v	2	novel enhancer binding protein, works in collaboration with AP1 and AP2
ETF	2	EGF transcription factor
AP-1	1	A retinoic acid-responsive transcription factor, inflammation

Supplementary Figure 11: Human RPE morphology before and after retinoic acid treatment.

The phase contrast images were taken by an ECHO Fluorescent Microscope (Thermo Fisher Scientific); 24 hour treatment with $10uM$ retinoic acid has no obvious impact on RPE cell morphology and viability. The experiment was repeated independently three times. Scale bar, 50µm.

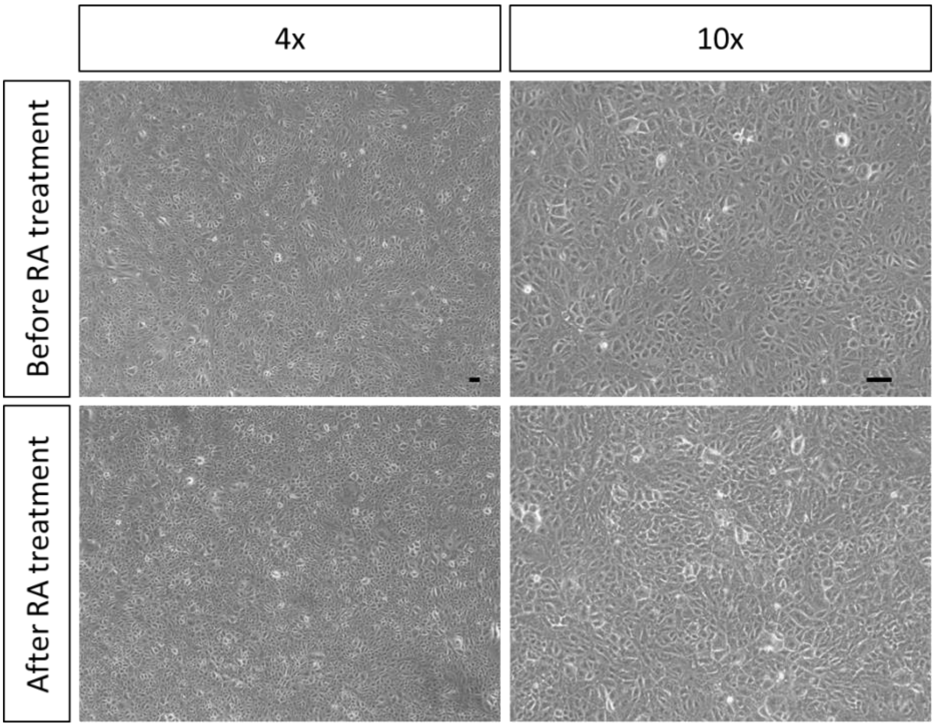

Supplementary Figure 12: mRNA expression in HRPE cells upon dexamethasone treatment.

Data are presented as the mean ± S.E.M. of three independent experiments. Statistical analysis was performed via a two-tailed, two-sample Student's *t*-test. Source data are provided in a Source Data file.

HRPE - human retinal pigment epithelial cells

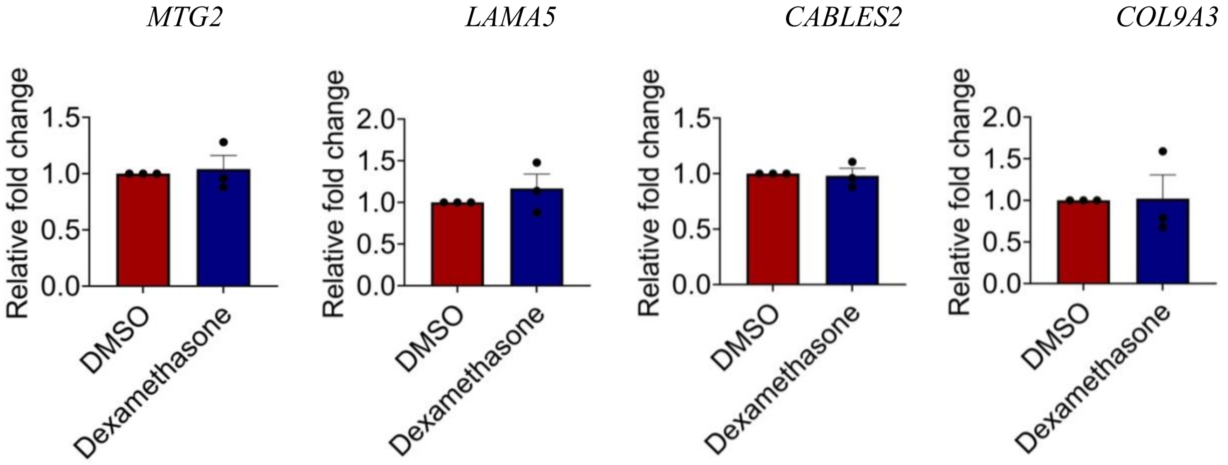

Supplementary Figure 13: The coverage of target samples in whole-exome sequencing.

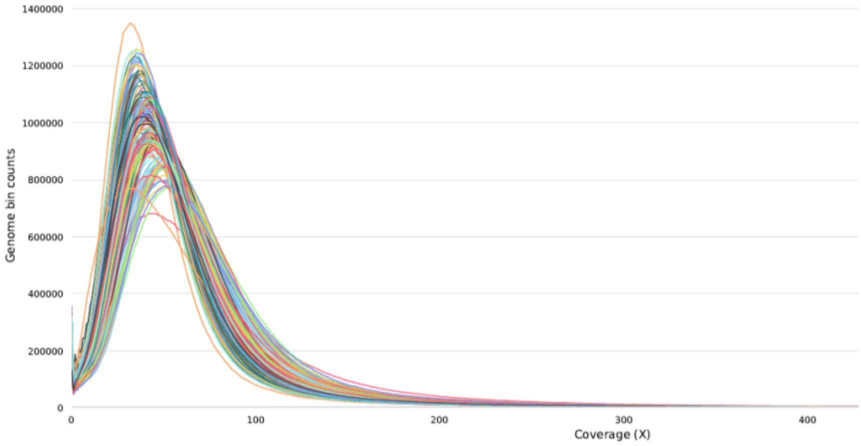

Batch 1

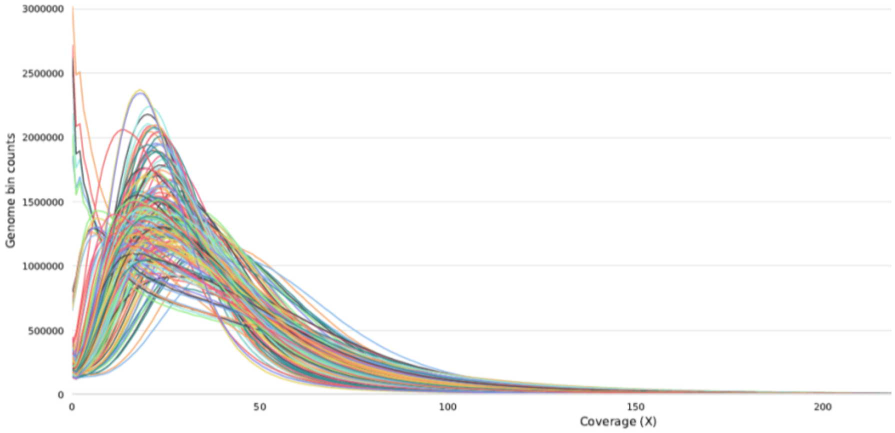

Batch 2

Supplementary Figure 14: Pair-wise LD heatmap at four novel loci in East Asians and Europeans.

Using genetic variants around 500 *kb* of the lead SNP and with *P*-value < 0.1 based on nAMD meta-analysis summary statistics.

GATA5 locus

East Asians | Europeans

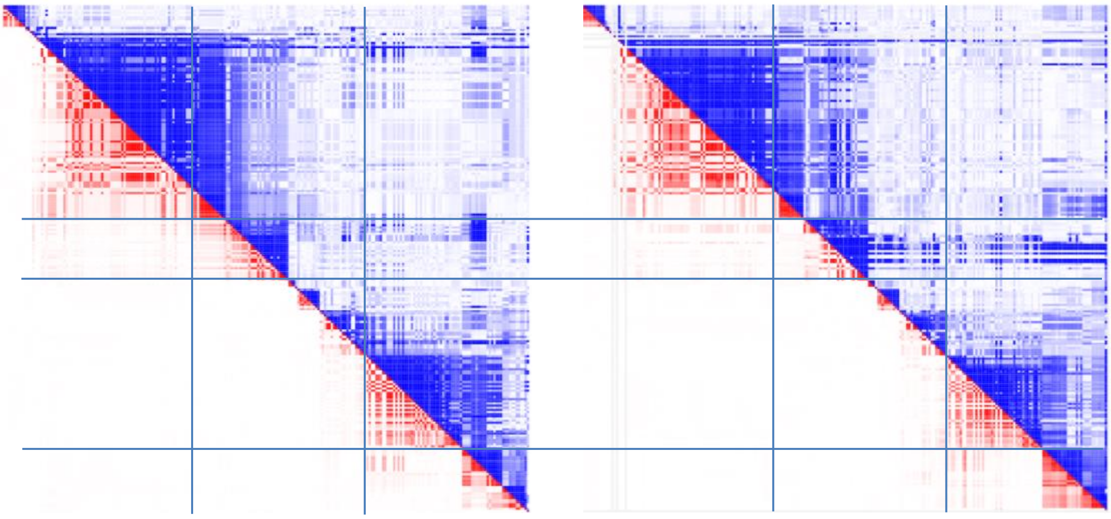

NEK6/LHX2 locus

East Asians | Europeans

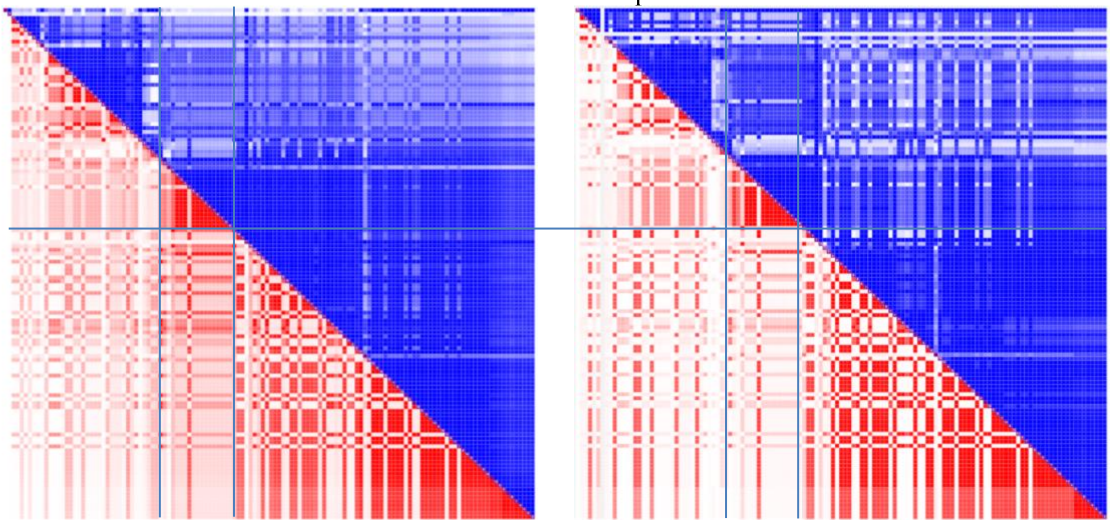

SPATA13 locus

East Asians

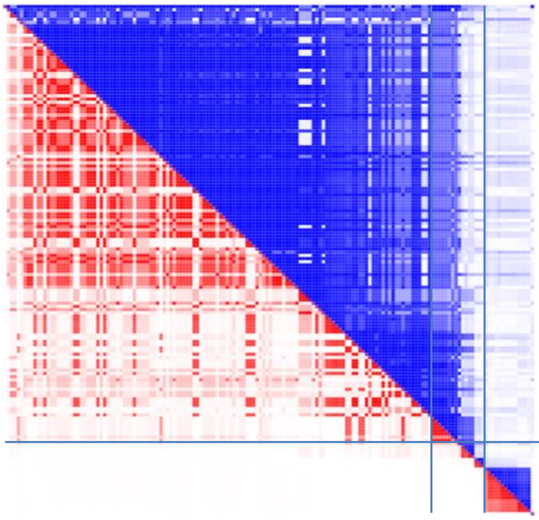

Europeans

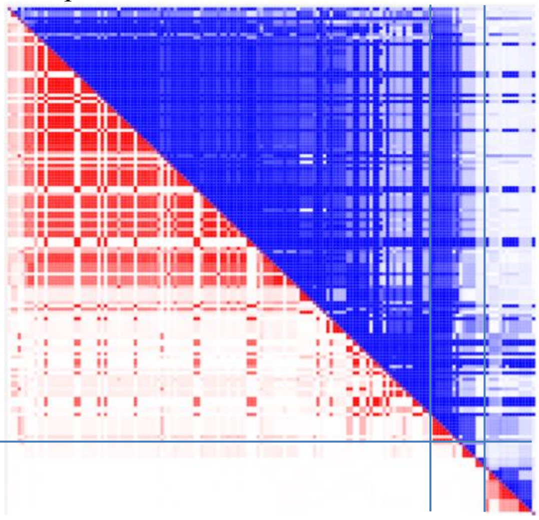

PCSK6 locus

East Asians

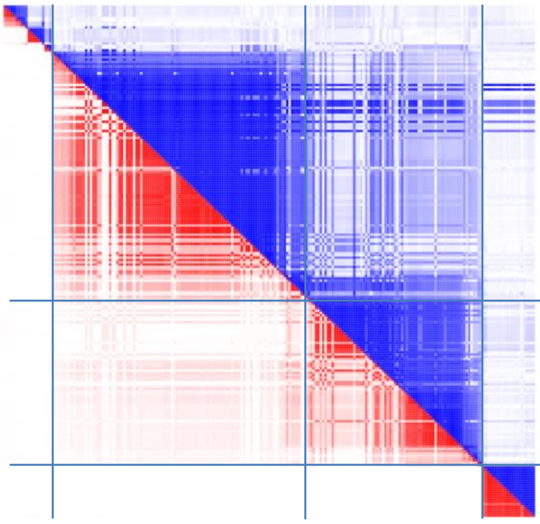

Europeans

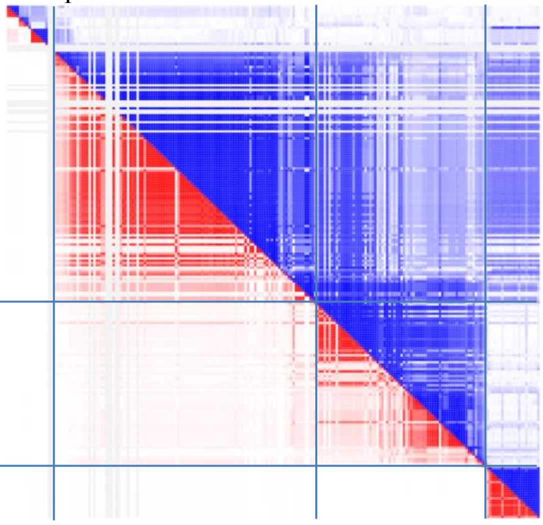

Supplementary References:

1. Cheung, C.M. *et al.* Asian age-related macular degeneration phenotyping study: rationale, design and protocol of a prospective cohort study. *Clin Exp Ophthalmol* **40**, 727-35 (2012).
2. Klein, R. *et al.* The Wisconsin age-related maculopathy grading system. *Ophthalmology* **98**, 1128-34 (1991).
3. Lavanya, R. *et al.* Methodology of the Singapore Indian Chinese Cohort (SICC) eye study: quantifying ethnic variations in the epidemiology of eye diseases in Asians. *Ophthalmic Epidemiol* **16**, 325-36 (2009).
4. Cheng, C.Y. *et al.* New loci and coding variants confer risk for age-related macular degeneration in East Asians. *Nat Commun* **6**, 6063 (2015).
5. Davis, M.D. *et al.* The Age-Related Eye Disease Study severity scale for age-related macular degeneration: AREDS Report No. 17. *Arch Ophthalmol* **123**, 1484-98 (2005).
6. Bei, J.X. *et al.* A genome-wide association study of nasopharyngeal carcinoma identifies three new susceptibility loci. *Nat Genet* **42**, 599-603 (2010).
7. Vithana, E.N. *et al.* Genome-wide association analyses identify three new susceptibility loci for primary angle closure glaucoma. *Nat Genet* **44**, 1142-1146 (2012).
