# Supplementary material for: Contribution of common and rare variants to Asian neovascular age-related macular degeneration subtypes
Source: Nat Commun. 2023 Sep 11;14:5574. doi: 10.1038/s41467-023-41256-z (PMC10495468; doi:10.1038/s41467-023-41256-z)
Supplement: Supplementary file 2 — Description of Additional Supplementary Files [file 41467_2023_41256_MOESM2_ESM.pdf]

Description of Additional Supplementary Files

Contribution of common and rare variants to Asian neovascular age-related macular degeneration subtypes

Qiao Fan et al

File Name: Supplementary Data 1
Description: Characteristics of age-related macular degeneration cases and controls among sample collections. PCV, polypoidal choroidal vasculopathy; typical nAMD, typical neovascular age-related macular degeneration; DFE, dilated fundus examination; FA, fluorescein angiography; ICG, indocyanine green angiography; OCT, optical coherence tomography; GWAS, genome-wide association study; WES, whole exome sequencing; No. of samples, the number of individuals who passed quality criteria.

File Name: Supplementary Data 2
Description: 2A. Association results for genetic loci at P-value $< 5 \times 10^{-5}$ in the GWAS discovery phase for neovascular AMD. 2B. Additional genetic loci at P-value 5×10^{-8} identified by a whole-genome meta-analysis of 4 cohorts. [a]SNP rs4643535 is genome-wide significant in meta-analysis for nAMD. It is independent with rs56033528 (LD $r^2 = 0$) at locus *RHBDD1/COL4A4* (the 8th loci in Supplementary Data 2A). [b]SNP rs72759285 at locus *NEK6/LHX2* is genome-wide significant (1.19×10^{-8}) in meta-analysis for typical nAMD. Locus, counts of loci; Chr, chromosome; EA, effect allele; OA, other allele; EAF, effect allele frequency. We conducted a clumping analysis using PLINK1.90 with parameter $r^2 = 0.05$, $250\ kb$, $P_1 = P_2 = 5 \times 10^{-5}$. Physical positions and nearest genes are based on NCBI build 37 of the human genome. The fixed effects model utilized the inverse-variance-weighted method. Random-effects model which corrects the deflation in the variance of the fixed-effects estimate employed the DerSimonian-Laird method.

File Name: Supplementary Data 3
Description: Additional independent variants conditioning on the lead variant, *CFH* rs800292 (chr1:196642233). We used GCTA to conduct the conditional and joint analysis (COJO). Variants flanking $500\ kb$ of the lead variants were included. Original GWAS data were used as a reference panel. Considering multiple comparisons, independent variants were defined as those with a P value $< 5 \times 10^{-8}$ in both AMD GWAS and conditional analysis. Physical positions and nearest genes are based on NCBI build 37 of the human genome. Chr, chromosome; MAF, minor allele frequency; Beta, effect size per copy of the minor allele; SE, standard error of Beta. Beta', SE' and P', effect size, standard error of effect size and P value from the conditional analysis.

File Name: Supplementary Data 4
Description: Gene-based analysis for neovascular AMD. We performed gene-based analysis using summary statistics, as implemented in FUMA. SNPs were mapped to the protein-coding genes with window sizes of 5 kb upstream and downstream of the gene. An SNP-wide mean model uses a test statistic that is equal to a mixture distribution of independent chi-square statistics with one degree of freedom, weighted by eigenvalues of the correlation matrix of the SNP genotypes. Simulation procedures were applied to generate empirical p-values. A total of 18,830 protein-coding genes based on Ensembl version 92 were included in the analysis. According to the Bonferroni correction method, we set a threshold of $0.05 / 18{,}830 = 2.66 \times 10^{-6}$ for significant genes.

File Name: Supplementary Data 5

Description: Evaluation of known AMD loci in the meta-analysis of Asian GWAS data. Summary statistics in European GWAS were obtained from the IAMDGC paper (PubMed ID: 26691988). The PubMed ID for Han's paper is 32277175. For the neovascular AMD GWAS, we examined 3,128 cases and 5,493 controls with East Asian ancestry. We applied a Firth logistic regression model in each cohort and later performed a fixed-effect meta-analysis across all cohorts. As rs72802342 (chr16:75234872) is not available in our GWAS, we replaced it by rs55993634 (chr16:75236763) which is in LD with the lead SNP (r2=0.93). As rs142450006 (chr20:44614991) is not available in our GWAS, we replaced it by rs1888235 (chr20:44623967) which is in LD with the lead SNP (r2=0.94). As rs2011092 (chr3:141124607) is not available in our GWAS, we replaced it by rs13066993 (chr3:141129999) which is in LD with the lead SNP (r2=0.99). As rs6565597 (chr17:79526821) is not available in our GWAS, we replaced it by rs9911460 (chr17:79538841) which is in LD with the lead SNP (r2=0.58). Chr, chromosome; EA, effect allele; OA, other allele; and OR, odds ratio based on effect allele.

File Name: Supplementary Data 6
Description: Evaluation of six loci in Table 1 in Europeans. We evaluated the effect size and significance of our lead variants in the international age-related macular degeneration genomics consortium (IAMDGC) study that contains 16,144 cases with advanced AMD and 17,832 controls of European ancestry. The IAMDGC study conducted a genome-wide single variant association analysis based on the Firth bias-corrected likelihood ratio test. We examined 3,128 cases with neovascular AMD and 5,493 controls of East Asian ancestry based on the same model. P-values were obtained from a chi-square distribution with one degree of freedom. Chr, chromosome; EA, effect allele; OA, other allele; Beta, association effect for per copy of the effect allele; SE, standard error of Beta; MAF, minor allele frequency; Z score, ratio of Beta to SE. Physical positions and nearest genes are based on NCBI build 37 of the human genome. § MAF based on the 1000 Genomes Phase 3 of European ancestry.

File Name: Supplementary Data 7
Description: Mapping rare variant burden in genes at GWAS loci. The rare variant burden was assessed by whole-exome gene-based analyses on functional variants at MAF $<$ 2%. MAC, total minor allele counts across functional variants in each gene. Ref1: Huang, L. et al. A missense variant in *FGD6* confers increased risk of polypoidal choroidal vasculopathy. Nature Genetics 48, 640-647 (2016). Ref2: Wen, X. et al. Association of *IGFN1* variant with polypoidal choroidal vasculopathy. The Journal of gene medicine 20, e3007 (2018).

File Name: Supplementary Data 8
Description: Functional variants in *ENTPD4* from WES PCV data. Three variants in bold (R591H, P202S, and L197V) are computationally predicted to be damaging (SIFT ≤ 0.05, PolyPhen2 ≥ 0.957 and CADD ≥ 20). WES analysis used variants at MAF $<$ 2%. Missense variants that had met the following criteria were computationally predicted to be damaging or deleterious: 1) probably damaging (D) in PolyPhen; 2) deleterious in SIFT (D) and 3) CADD > 20. SIFT_pred: D for Deleterious (SIFT_score ≤ 0.05) and T for tolerated (SIFT_score > 0.05). Polyphen2_pred: D for Probably damaging (Polyphen2_score ≥ 0.957), P for possibly damaging ($0.453 \leq$ Polyphen2_score ≤ 0.956); B for benign (Polyphen2_score ≤ 0.452).

File Name: Supplementary Data 9
Description: Association of functional variants flanking $500\ kb$ of 12 GWAS loci for PCV. A total of 320 rare and low-frequent variants (MAF $<$ 5% & MAF $>$ 0.1%) were included. Single variants at P-value < 0.05 are listed in the table. The significance at P-value $< 0.05\ /\ 320 = 1.56 \times 10^{-3}$ after multiple testing correction.

File Name: Supplementary Data 10
Description: 10A. WES gene-based analyses for PCV on functional variants at MAF $<$ 2%. We conducted gene-based analyses (SKAT-O, SKAT, and Burden test) to investigate the role of functional

variants at MAF $< 2\%$ for PCV. The analysis included a total of 1,019 subjects (259 cases and 760 controls). All subjects are of Chinese descent from Singapore. The kernel-regression-based association tests were adjusted on age, gender and the top 4 principal components. We presented genes with a P-value cutoff of $< 1 \times 10^{-3}$. Based on the Bonferroni correlation method, the P-value threshold of significant genes (highlighted in bold) was set as 0.05 / $16{,}794$ genes $= 2.98 \times 10^{-6}$. The significant gene was highlighted in bold. 10B. WES gene-based analyses for PCV on functional variants at MAF $< 1\%$. We conducted gene-based analyses (SKAT-O, SKAT and Burden test) to investigate the role of functional variants at MAF $< 1\%$ for PCV. The analysis included a total of 1,019 subjects (259 cases and 760 controls). All subjects are of Chinese descent from Singapore. The kernel-regression-based association tests were adjusted on age, gender and the top 4 principal components. We presented genes with a P-value cutoff of $< 1 \times 10^{-3}$. Based on the Bonferroni correction method, the P-value threshold for significant genes was set at $0.05 / 16{,}765 = 2.98 \times 10^{-6}$. The significant gene was highlighted in bold. The R package "SKAT" uses six methods (method.bin) to compute P-values: 1) Efficient resampling (ER); 2) Quantile adjusted moment matching (QA); 3) Moment matching adjustment (MA); 4) No adjustment (UA); 5) Adaptive ER (ER.A); and 6) Hybrid. "Hybrid" selects a method based on the total minor allele count (MAC), the number of individuals with minor alleles (m), and the degree of case-control imbalance. When method.bin="ER" or "ER.A", SKATBinary computes mid-P-values and minimum achievable mid-P-values. N.Marker.All, the number of SNPs in the genotype matrix; N.Marker.Test, the number of SNPs used for the test; MAC, total minor allele count (MAC); m, the number of individuals with minor alleles; Method.bin, a type of method to be used to compute the p-value; MAP, minimum possible P-values. It is available when the method.bin="ER" and m is sufficiently small. 1 or -1 represents no MAP was computed.

File Name: Supplementary Data 11
Description: Functional variants in *COL1A1* for WES PCV dataset. 10 variants in bold computationally predicted to be damaging (SIFT ≤ 0.05, PolyPhen ≥ 0.957 and CADD ≥ 20). WES analysis included variants at MAF $< 2\%$.

File Name: Supplementary Data 12
Description: Top loci from GWAS of PCV versus typical nAMD. The lead variant was identified from genome-wide single-variant meta-analysis for PCV versus typical nAMD, defined by the one with the smallest P-value at a locus. The analysis included 1,555 patients with PCV as cases and 1,573 patients with typical nAMD as controls. Physical positions and nearest genes are based on NCBI build 37 of human genome. Chr, chromosome; Beta, effect for per copy of the minor allele; SE, standard error of Beta; Minor allele is effect allele for Beta. P_dif shows the significance of the different of Zscore between PCV GWAS and typical nAMD GWAS.

File Name: Supplementary Data 13
Description: Summary of implicated genes for AMD, neurovascular AMD and PCV, from GWAS and WES. Nearest genes at GWAS top loci, or genes identified from WES[41,63-74]. The genes identified from the current study are also included.

File Name: Supplementary Data 14
Description: Genome-wide pathway analysis for neovascular AMD and subtypes. A total of 15,481 pathways including Curated gene sets and GO terms were used, as implemented in MAGMA. Bonferroni corrected P-value is 3.23×10^{-6}. All pathways reached a P-value threshold being 1×10^{-4} were provided below. The significant pathway was highlighted in bold. NGENES: number of genes in a pathway.

File Name: Supplementary Data 15
Description: Corresponding genes involving in top pathways for PCV and their association P-value in gene-based analysis. SNPs within a 5 kb window were assigned to a gene. Gene-based analysis was conducted using GWAS summary statistics.

File Name: Supplementary Data 16
Description: eQTLs mapping for novel genetic loci using GTEx v8 cis-eQTL associations. We mapped 4 novel lead SNPs at *GATA5*, *NEK6*, *SPATA13*, and *PCSK6* loci to GTEx v8 cis-eQTL datasets. We reported SNP-gene pairs with P-value $< 5 \times 10^{-3}$. The significance of the cis-eQTL is claimed after multiple testing at P-value $= 0.05 / 53 = 9.43 \times 10^{-4}$.

File Name: Supplementary Data 17
Description: Sequences of the forward and reverse primers utilized for gene expression analysis.
