# Supplementary material for: Contribution of common and rare variants to Asian neovascular age-related macular degeneration subtypes
Source: Nat Commun. 2023 Sep 11;14:5574. doi: 10.1038/s41467-023-41256-z (PMC10495468; doi:10.1038/s41467-023-41256-z)
Supplement: Supplementary file 4 — Reporting Summary [file 41467_2023_41256_MOESM4_ESM.pdf]

nature portfolio

Corresponding author(s): Qiao Fan

Last updated by author(s): Aug 3, 2023

Reporting Summary

Nature Portfolio wishes to improve the reproducibility of the work that we publish. This form provides structure for consistency and transparency in reporting. For further information on Nature Portfolio policies, see our Editorial Policies and the Editorial Policy Checklist.

Statistics

For all statistical analyses, confirm that the following items are present in the figure legend, table legend, main text, or Methods section.

n/a	Confirmed	
☐	☒	The exact sample size (*n*) for each experimental group/condition, given as a discrete number and unit of measurement
☐	☒	A statement on whether measurements were taken from distinct samples or whether the same sample was measured repeatedly
☐	☒	The statistical test(s) used AND whether they are one- or two-sided<br>*Only common tests should be described solely by name; describe more complex techniques in the Methods section.*
☐	☒	A description of all covariates tested
☐	☒	A description of any assumptions or corrections, such as tests of normality and adjustment for multiple comparisons
☐	☒	A full description of the statistical parameters including central tendency (e.g. means) or other basic estimates (e.g. regression coefficient) AND variation (e.g. standard deviation) or associated estimates of uncertainty (e.g. confidence intervals)
☐	☒	For null hypothesis testing, the test statistic (e.g. *F*, *t*, *r*) with confidence intervals, effect sizes, degrees of freedom and *P* value noted<br>*Give P values as exact values whenever suitable.*
☒	☐	For Bayesian analysis, information on the choice of priors and Markov chain Monte Carlo settings
☒	☐	For hierarchical and complex designs, identification of the appropriate level for tests and full reporting of outcomes
☐	☒	Estimates of effect sizes (e.g. Cohen's *d*, Pearson's *r*), indicating how they were calculated

Our web collection on statistics for biologists contains articles on many of the points above.

Software and code

Policy information about availability of computer code

Data collection	Each study collected data individually with the method/software described in the supplementary file.
Data analysis	GWAS genotyping data quality control: PLINK 1.90 beta, R 3.5.3, EIGENSOFT v7.2.1<br>Imputation: Minimac4 on the Michigan Imputation Server<br>GWAS analysis, meta-analysis, and post-GWAS analysis: EPACTS v3.3.0, METAL (the version released on March 2011), GWAMA v2.2.2, FUMA v1.3.6a, ANNOVAR (the version released on 8th June 2020), GCTA 1.92.4 beta version, and LocusZoom v1.4.<br>WES data quality control: BWA v0.7.10 and GATK v4.03<br>Association analysis of rare variants: EPACTS v3.3.0, R package “SKAT” v2.0.1<br>Heritability and genetic correlation: LDSC v1.0.1<br>In silico analysis of transcription factor binding sites: PROMO v8.3 which incorporates TRANSFAC v6.4 and FunciSNP.<br>Statistical Analyses in Animal study: Prism 8.0

For manuscripts utilizing custom algorithms or software that are central to the research but not yet described in published literature, software must be made available to editors and reviewers. We strongly encourage code deposition in a community repository (e.g. GitHub). See the Nature Portfolio guidelines for submitting code & software for further information.

Data

Policy information about availability of data

All manuscripts must include a data availability statement. This statement should provide the following information, where applicable:

- Accession codes, unique identifiers, or web links for publicly available datasets
- A description of any restrictions on data availability
- For clinical datasets or third party data, please ensure that the statement adheres to our policy

The summary statistics of the top index variants were presented in the Supplementary Data, along with the data analyzed in this study. The summary statistics file from the meta-analysis for nAMD is available on the Amazon Web Services (AWS) from https://gamagentics.s3.ap-southeast-1.amazonaws.com/AMD_Topvariants_EAS_NC2023.txt. Other datasets generated during the study can be obtained by contacting the corresponding authors. The GWAS results for advanced AMD that we used in this study are available on the GWAS Catalog http://ftp.ebi.ac.uk/pub/databases/gwas/summary_statistics/GCST003001-GCST004000/GCST003219/ under study accession identifier GCST003219. The 1000 Genomes Phase 3 data on Genome Reference Consortium Human Build 37 (GRCh37) is available at https://www.internationalgenome.org/. The datasets used for eQTL mapping and gene-set analysis through the FUMA platform (https://fuma.ctglab.nl/) are available through: GTEx eQTL v8 and EyeGEx (https://www.gtexportal.org/home/datasets) and MSigDB v7.0 gene-set file (https://www.gsea-msigdb.org/gsea/msigdb). Source data supporting our findings (Figure 3 and Supplementary Figures 8,9, and 12) were provided in this paper as a Source Data file.

Human research participants

Policy information about studies involving human research participants and Sex and Gender in Research.

Reporting on sex and gender	We did not report sex/gender-specific results. We accounted for sex as a covariate in the whole-exome sequencing (WES) analysis.
Population characteristics	The population characteristics of each contributing study for the genome-wide association studies (GWAS) has been provided in the Supplementary Information and Supplementary Data 1. The WES data analysis included 1,019 individuals (259 cases with PCV and 760 controls) of the Chinese ancestry. Details were provided in the Method section in the paper.
Recruitment	In this study, the GWAS analysis recruited 3,128 cases with neovascular age-related macular degeneration (nAMD) and 5,493 controls of the East Asian ancestry from 4 independent studies. The number of nAMD cases, two clinical subtypes, and controls for each sample collection, as well as genotyping information were provided in the Supplementary Data 1. Our WES analysis included 259 polypoidal choroidal vasculopathy (PCV) cases and 760 controls of the Chinese ancestry. All participating studies used similar protocols for nAMD and subtypes measures and details were provided in the Supplementary Information.
Ethics oversight	All studies were performed with the approval of their Human Research and Ethics Committee, adhering to the Declaration of Helsinki principles. Written informed consent was obtained by the ethics committee of all the participating institutions as follows:<br>Singapore: Singapore National Eye Center, National University Health System, Tan Tock Seng Hospital, and the Centralized Institutional Review Board (CIRB).<br>Hong Kong: Hong Kong Eye Hospital, Prince of Wales Hospital Eye Center, Prince of Wales Hospital, and Sun Yat-sen University Cancer Center.<br>Japan: Department of Ophthalmology at Kyoto University Hospital, Fukushima Medical University Hospital, Kobe City Medical Center General Hospital, Ozaki Eye Hospital, Mizoguchi Eye Clinic, Japanese Red Cross Otsu Hospital, and Nagahama City Hospital.<br>Korea: Seoul National University Bundang Hospital, Seoul National University Hospital, Kyungpook National University Hospital, Yeungnam University Hospital, Kosin University Hospital, and Busan Paik Hospital.

Note that full information on the approval of the study protocol must also be provided in the manuscript.

Field-specific reporting

Please select the one below that is the best fit for your research. If you are not sure, read the appropriate sections before making your selection.

☒ Life sciences ☐ Behavioural & social sciences ☐ Ecological, evolutionary & environmental sciences

For a reference copy of the document with all sections, see nature.com/documents/nr-reporting-summary-flat.pdf

Life sciences study design

All studies must disclose on these points even when the disclosure is negative.

Sample size	For GWAS analysis, we included all available samples to boost power as genetic effects of common variants are moderate. We collected 3,128 cases and 5,493 controls of the East Asians from the 4 independent sample collections.
Data exclusions	Pre-imputation QC for GWAS: The centralized quality control (QC) procedure for GWAS genotyping data was applied to each study. Autosomal

Data exclusions	variants with minor allele frequency (MAF) <0.5%, genotype call rates <95%, or deviation from Hardy-Weinberg equilibrium (HWE) (P<1E-6) were excluded. Participants with Identity-by-decent (IBD) >0.1875, missing genotype calls >5% or an excess of heterozygosity (>6 SD) were excluded. We only kept individuals of the East Asian ancestry. We further excluded palindromic variants (A/T or G/C) with MAF >0.4 and variants with an allele frequency difference >0.2 in our data and the 1000 Genomes Phase 3 reference data. All variants were aligned to the forward strand, and reference alleles were fixed according to the reference panel. For more details, please see Method section in the main text.<br><br>Post-imputation QC for GWAS: Genetic variants of imputation quality ≥0.5 in at least 3 studies were included in the association analysis.<br><br>QC for WES: Before applying GATK to call genotypes, the adaptors and sites with a low Phred score (<20) were removed. Genotypes were first filtered by the GATK variant quality score recalibration (VQSR). Additional filters excluded the sites with low depth rates (<10×), high depth rates (>300×), excessive heterozygosity (P<1E-6), HWE P-value <1E-4, or missingness rate >10%. We excluded 37 controls with early AMD, three typical nAMD patients and one Malay case. We further excluded 38 cases with a missingness rate of genotypes >10%. Variants failed to pass the batch effect check were also excluded.
Replication	We validated and replicated our findings in Korean and Japanese samples. The replication results of top variants during the discovery phase which have a P-value <5E-5 were provided in the Supplementary Data 2A.
Randomization	Samples recruited for this study were not randomized. To account for potential confounding of population stratification, we adjusted top principal components in the association tests. We assessed genomic inflation by using genomic control factor (λ).
Blinding	Blinding is not applicable to GWAS study.

Reporting for specific materials, systems and methods

We require information from authors about some types of materials, experimental systems and methods used in many studies. Here, indicate whether each material, system or method listed is relevant to your study. If you are not sure if a list item applies to your research, read the appropriate section before selecting a response.

Materials & experimental systems

n/a	Involved in the study
☒	☐ Antibodies
☒	☐ Eukaryotic cell lines
☒	☐ Palaeontology and archaeology
☐	☒ Animals and other organisms
☒	☐ Clinical data
☒	☐ Dual use research of concern

Methods

n/a	Involved in the study
☒	☐ ChIP-seq
☒	☐ Flow cytometry
☒	☐ MRI-based neuroimaging

Animals and other research organisms

Policy information about studies involving animals; ARRIVE guidelines recommended for reporting animal research, and Sex and Gender in Research

Laboratory animals	C57BL/6J mice were purchased from InVivos Pte Ltd., Singapore. All mice were housed in an environmentally controlled room (22°C, 40-60% humidity, and a 12-h light cycle) with food and water available ad libitum.
Wild animals	Not applicable
Reporting on sex	mixed gender
Field-collected samples	ocular retina tissues
Ethics oversight	All experiments using animals were approved by the Institutional Animal Care and Use Committee of the Agency for Science, Technology, and Research (A*STAR) (IACUC, Protocol number: 181334) and SingHealth Experimental Medicine Centre (SEMC) (IACUC, Protocol number: 2018/SHS/1449) in Singapore. Animal care and procedures were performed in accordance with the Guide for Care and Use of Laboratory Animals from the US National Institutes of Health and the Statement for the Use of Animals in Ophthalmic and Vision Research from the Association for Research in Vision and Ophthalmology (ARVO).

Note that full information on the approval of the study protocol must also be provided in the manuscript.
